# Relative risk and excess fraction of preterm birth across maternal occupation and industry: a Danish nationwide register-based cohort study of employed pregnant women

**DOI:** 10.5271/sjweh.4310

**Published:** 2026-09-01

**Authors:** Astrid Juhl Andersen, Marie Pedersen, Harald Hannerz, Sandra Søgaard Tøttenborg, Esben Meulengracht Flachs, Jens Peter Ellekilde Bonde, Jenny Selander, Luise Mølenberg Begtrup, Camilla Sandal Sejbaek, Karin Sørig Hougaard

**Affiliations:** 1 National Research Centre for the Working Environment, Copenhagen, Denmark.; 2 Section of Environmental Health, Department of Public Health, University of Copenhagen, Copenhagen, Denmark.; 3 Department of Occupational and Environmental Medicine, Copenhagen University Hospital – Bispebjerg and Frederiksberg, Copenhagen, Denmark.; 4 Department of Public Health, University of Copenhagen, Copenhagen, Denmark.; 5 Institute of Environmental Medicine, Karolinska Institutet, Stockholm, Sweden.

**Keywords:** Denmark, DOC*X Generation, maternal employment, premature birth, registry study

## Abstract

**Objectives:**

Occupational exposures can increase the risk of preterm birth (PTB). We hypothesized that the risk varies significantly according to maternal work tasks and aimed to quantify relative risks and excess fractions of PTB related to occupation and industry during pregnancy.

**Methods:**

This nationwide study of 644 349 singleton pregnancies (2004–2018), classified maternal employment the year of conception into 38 occupational and 39 industrial groups. To approximate the lowest risk and excess fraction of PTB, ie, live birth before 37 completed gestational weeks, we constructed simulation-based reference groups that accounted for within-group random variation. Adjusted risk ratios (RR) and 99% confidence intervals (CI) of PTB were estimated relative to the simulation-based lowest risk reference and the population average.

**Results:**

Increased risks of PTB were observed for 22 occupational and 10 industrial groups, compared with the simulation-based lowest risks. The highest RR were found for assembly workers (1.42, 99% CI 1.12–1.79) and home care workers (1.35, 99% CI 1.24–1.47). For industries, the highest RR were found for agriculture, forestry and fishing (1.29, 99% CI 1.06–1.57) and manufacture of metals and machinery (1.22, 99% CI 1.07–1.40). Compared to the population average, risks were significantly higher for home care workers, nursing home workers, clerks (not elsewhere classified), shop assistants, and jobs with unstated job codes, and for the industrial groups of manufacture of metals and machinery and residential centers and home help.

**Conclusion:**

Several maternal occupational and industrial groups in Denmark had elevated risks of PTB. The findings are exploratory and further research is needed to identify the potential for prevention in the occupational setting.

Preterm birth (PTB), defined as delivery before 37 weeks of gestation, is a leading cause of infant mortality and various pediatric and chronic diseases (1–5). While the global prevalence is around 11%, it is only 5–6% in the Nordic countries, including Denmark (4, 6). Several occupational hazards have been linked to increased risk of PTB, such as physical workload, including prolonged standing and heavy lifting, whole-body vibration, psychosocial work conditions, long working hours, shift work, heat and exposure to some chemicals (7–15). There is a substantial gap in the understanding of mechanisms underlying PTB, which can best be described as a syndrome in which the final phenotype arises from multiple etiologies, that is, activation of several processes may prematurely trigger components of the common pathway to labor (16, 17). This gap extends to the potential mechanisms by which different occupational risk factors may lead to PTB.

In Denmark and the EU, it is a legislative requirement that pregnant women must be able to work without risking harm to their pregnancy or unborn child (18). Previous studies have predominantly used single-factor approaches in the study of occupational risk factors for PTB, which do not provide insight into the overall risk associated with jobs involving multiple concurrent occupational exposures. To obtain a less biased and broader understanding of risks across employment groups, we aimed to study the risk of PTB across occupations and industries in Denmark. This approach allows consideration of the combined influence of various factors within groups and provides a comprehensive overview that can help identify and compare occupational and industrial groups with different risk profiles, enabling identification of groups with increased risks and potential for prevention. This can be used to guide future studies of workplace interventions and the efficiency of implemented guidelines intended to protect pregnant employees.

In Denmark, around 60 000 children are born every year (19), mostly to mothers working before and during pregnancy. In a nationwide study of all women with registered employment in Denmark in the year of conception who delivered live-born singletons from 2004 to 2018, we aimed to quantify the relative risk of PTB associated with major maternal occupational and industrial groups during pregnancy. Furthermore, we aimed to estimate the excess fractions related to occupational and industrial groups to assess the potential for proportional reduction in PTBs if all pregnancies had the same risk level as those in the occupational or industrial groups with the lowest risk.

## Methods

This study was conducted according to a prespecified study protocol (20). Deviations from the study protocol that was agreed upon prior to initiation of the analyses are described in the supplementary material (XXX) S1.

### Data material and study population

**Figure 1:**
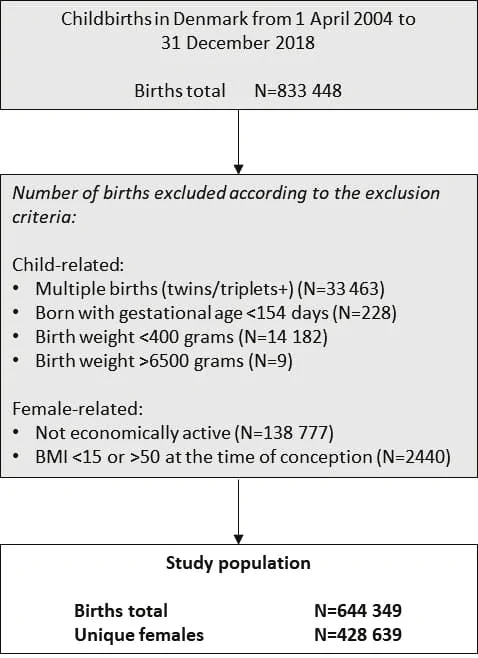
Flowchart.

In this register-based cohort study, we used a subset of the national pregnancy cohort, DOC*X-Generation (DOC*X-G), an extension of the Danish Occupational Cohort with eXposure (DOC*X). The DOC*X is an open cohort covering all employees aged ≥16 years living in Denmark, who had ≥1 year of gainful employment in 1970 or from 1976 onwards (21, 22). We included all women giving birth to live-born singletons, between 1 April 2004 and 31 December 2018, with a gestational age of 22–45 weeks and a birth weight of 400–6500 grams. The start date was chosen based on the change in definition of clinical abortion on 1 April 2004 in Denmark, from termination of pregnancy before 28 to before 23 completed weeks of gestation. The women had to be economically active, ie, registered with an occupation in the calendar year of the estimated date of conception. Date of conception was based on registered gestational age, assessed by midwives’ or doctors’ clinical registration, date of last menstrual period, or ultrasound estimation. Women with a recorded pre-pregnancy body-mass index (BMI) <15 and >50 kg/m^2^ were excluded due to the disproportionately higher risks of gestational health complications, including PTB, in these groups (figure 1). Information on conception date, gestational age, pre-pregnancy BMI, smoking during pregnancy, parity, season of conception and calendar period of birth was retrieved from the Danish Medical Birth Register (23), information on employment from the Employment Classification Module (24) and information on marital status and migration from the Central Person Register (25).

### Ethical approval

The cohort is register-based, and therefore no approval from the Danish National Committee on Health Research was required. Permission to undertake the study was obtained from the Regional Data Protection Authority (P-2019-217).

### Exposure: occupation and industry

We used information on the women’s main employment in the calendar year of estimated conception. The Employment Classification Module contains 372 different job groups registered with DISCO-88 codes (the Danish version of ISCO-88). Based on knowledge on work tasks and the numbers of employed pregnant women, job codes were grouped into 38 occupational groups as described and listed in Begtrup et al (26) and the study protocol (20). The 726 industry codes in the Danish Industry Classification 2007 (DB07) were aggregated into 39 industrial groups according to the Danish Working Environment Authority. We conducted separate analyses for occupational and industrial groups.

### Outcome: preterm birth

PTB was defined based on gestational age as any births occurring from 22–36 completed weeks of gestation, compared with full-term and post-term births (37–45 completed gestational weeks). The lower limit is based on the threshold for spontaneous abortion (27). In sensitivity analyses, PTB was additionally categorized as very preterm (22–31 gestational weeks; extremely and very preterm were combined due to few cases), moderately preterm (32–36 weeks) and term and post-term birth (37–45 weeks) (5).

### Covariates

Potential confounders were identified *a priori* using a directed acyclic graph (supplementary figure S1) and previous literature. They included maternal age (<25, 25–29, 30–34, ≥35 years); country of origin (Denmark, other European countries, outside Europe or unstated); marital status (married, registered partner or cohabitant, single or unstated); pre-pregnancy BMI (15–18.4, 18.5–24.9, 25–29.9, 30–50 kg/m^2^ or unstated); smoking during pregnancy (yes/no or unstated); parity at conception (0, 1, ≥2); conception quarter of the year (Q1: January–March, Q2: April–June, Q3: July–September, Q4: October–December); calendar period of birth (1 April 2004–31 December 2008, 2009–2013, 2014–2018); child sex (female/male); maternal socioeconomic status (SES) at conception, measured in accordance with the occupationally based three level version of the European Socioeconomic Classification (low, medium, high or unstated) (28) with few adjustments; and household income the year prior to conception, indexed to 2018 values in standardized income for the whole study period (≤300 000, 300 001–500 000, 500 001–700 000, 700 001–900 000, ≥900 001 DKK or unstated).

### Statistical analysis

We examined associations between maternal occupational and industrial group and PTB using logistic regression models with generalized estimating equations to account for repeated measurements (>1 births per woman during the study period) to calculate expected numbers of PTB in each group. Analyses were conducted separately for occupation and industry. See supplementary figure S2 for an overview of the three statistical models described below. Model 1 included minimal adjustment for maternal age, parity, conception quarter, calendar period of birth, and infant sex. Model 2 included the main model, added adjustment for country of origin, marital status, pre-pregnancy BMI, and smoking during pregnancy. In the industry analyses, maternal SES was additionally included to account for variations in SES across industries. (This was not performed for the job groups analyses due to the occupational basis of the applied SES classification). Logistic regression was used to model the odds of each of the birth outcomes as a function of occupational and industrial groups. Adjusted parameter estimates from the logistic regression models were then used to calculate the expected number of PTB in each occupational or industrial group under the null hypothesis of no between-group differences. These calculated numbers of PTB were thereafter used to estimate risk ratios (RR), as the ratio of the observed to the expected numbers of PTB for each group and 99% confidence intervals (CI) were constructed to account for multiple comparisons. We applied 99% CI over 95% CI as hundreds of rate ratios were reported for this study, thereby reducing the likelihood that too many estimated CI would be interpreted as statistically significant due to type I error.

We then estimated excess fractions, defined as the proportion of cases that would not have occurred if all groups had the same risk as the lowest-risk group. Excess fraction is one of several terms used for this concept; another commonly used term is population attributable fraction (29). To approximate the lowest risk and the corresponding excess fraction, we applied a Monte Carlo simulation procedure that accounted for within-group random variation. Using the simulation-based lowest-risk group as the reference, we subsequently estimated group-specific fully adjusted RR and 99% CI under the hypothesis that the risk of PTB depends on occupational/industrial group (model 3).

In sensitivity analyses, we estimated the RR for very preterm and moderately preterm births using the main model 2. Additional sensitivity analyses included: (i) restricting the population to each woman’s first birth during the study period to avoid counting several births to the same women, often while she remained in the same occupational/industrial group; (ii) additional adjustment for household income the year prior to conception as an additional indicator of socio-economic status; (iii) stratification by time period (1 April 2004–31 December 2011 and 2012–2018) to assess temporal changes; and (iv) restriction to women of Danish origin to account for differences in risk by ethnicity. Sensitivity analyses were conducted for both occupational and industrial groups in subsets from of the total population.

All analyses were conducted in SAS 9.4 (SAS Institute, Cary, North Carolina, USA).

## Results

### Characteristics of study population

We included 644 349 pregnancies with live-born singletons from 428 639 occupationally active women including 30 167 cases of PTB in Denmark during 2004–2018. Most women were aged 25–34 years (71%), originated from Denmark (88%), and were married (76%) (table 1). Some differences in sociodemographic characteristics were observed across occupational and industrial groups (supplementary material S3, tables S1 and S2). Among occupational groups, the highest proportion of women born outside Europe or with unstated origin was found among cleaners, janitors and kitchen helpers (24.9% versus 4.9% overall). A pre-pregnancy BMI ≥30 kg/m^2^ and smoking during pregnancy were most common among home care workers (21.0% versus 11.1% overall and 26.7% versus 10.0% overall, respectively). Among industries, the cleaning industry had the highest proportion of women born outside of Europe or with unstated origin (25.9%), residential centers and home help had the highest proportion of women with BMI ≥30 kg/m^2^ (18.2%), while smoking during pregnancy was most common in the building completion and finishing industry (21%).

**Table 1:** Study population characteristics (N=644 349).

		N ^b^	%
Age (years) ^a^			
	<25	73 518	11.4
	25–29	227 050	35.2
	30–34	233 123	36.2
	≥35	110 658	17.2
Country of origin ^a^			
	Denmark	566 289	87.9
	Europe	46 642	7.2
	Outside of Europe or unstated	31 418	4.9
Marital status ^a^			
	Married	490 279	76.1
	Registered partner or cohabitant	822	0.1
	Single	140 407	21.8
	Unstated	12 841	2.0
Maternal socioeconomic status ^a^			
	High	227 757	35.4
	Intermediate	162 594	25.2
	Low	201 203	31.2
	Unstated	52 795	8.2
Pre-pregnancy body mass index (kg/m^2^)			
	15–18.4	23 639	3.7
	18.5–24.9	397 305	61.6
	25–29.9	130 745	20.3
	30–50	71 964	11.2
	Unstated	20 696	3.2
Maternal smoking during pregnancy			
	Yes	64 157	10.0
	No	567 141	88.0
	Unstated	13 051	2.0
Parity at conception			
	0	307 273	47.7
	1	240 610	37.3
	≥2	96 466	15.0
Conception quarter (Q)			
	Q1	154 537	24.0
	Q2	153 645	23.8
	Q3	162 726	25.3
	Q4	173 441	26.9
Calendar period of birth			
	1 April 2004–2011	347 153	
	2012–2018	347 118	33.2
Sex of child			
	Female	313 166	48.6
	Male	331 183	51.4

^a^ Measured the year of conception. ^b^ N refers to numbers of births.

### Occupation and PTB

Overall, occupational group was associated with PTB (table 2). In the minimally adjusted model 1, home care workers, clerks (not elsewhere classified), nursing home workers, shop assistants, and those with unstated job codes had statistically significantly higher risk of PTB compared with the average population risk. In contrast, medical doctors, dentists, and veterinarians, teachers in primary school and in higher, secondary, vocational, and special education, professionals (physics, mathematics, engineering, and architects) and professionals not elsewhere classified had lower risk. Results from the main model 2 were consistent with those of the minimally adjusted model 1, albeit with slightly lower RR.

**Table 2:** Number of births (N), observed number of cases of preterm birth, risk ratios (RR), and excess cases with 99% confidence interval (CI) by occupational group, ordered by social class and alphabetically within social class (total N=644 349).

Occupational group and social class ^a^	N	Observed cases	Model 1 ^b^			Model 2 ^c*^			Model 3 ^d*^			Excess cases ^d*^	
			RR	99% CI		RR1	99% CI		RR2	99%CI		Cases	99% CI
1. Medical doctors, dentists, and veterinarians	12 537	435	0.76	0.68–0.85		0.79	0.69–0.91		0.91	0.79–1.05		-40.9	-103.7–22.0
1. Professionals (physics, mathematics, engineering, architects)	15 350	613	0.83	0.76–0.92		0.85	0.77–0.95		0.98	0.87–1.11		-9.6	-82.8–63.7
1. Professionals at academic level, not elsewhere classified	54 556	2229	0.85	0.81–0.90		0.87	0.83–0.92		1.01	0.93–1.09		18.2	-149.9–186.2
1. Senior officials and corporate managers	6035	285	0.98	0.84–1.14		0.99	0.85–1.15		1.14	0.97–1.34		34.9	-10.5–80.4
2. Associate professionals (business and administration)	51 144	2325	0.95	0.90–1.00		0.96	0.91–1.01		1.11	1.03–1.19		227.7	62.3–393.1
2. Associate professionals (physics and engineering)	10 065	433	0.91	0.81–1.02		0.92	0.81–1.04		1.06	0.92–1.21		23.2	-35.2–81.7
2. Managers (<10 employees)	2247	112	1.06	0.83–1.37		1.05	0.82–1.34		1.21	0.94–1.55		19.4	-8.2–47.1
2. Psychologists and social workers	10 062	444	0.96	0.85–1.08		0.97	0.86–1.10		1.12	0.98–1.28		47.4	-11.9–106.7
2. Teachers in higher, secondary, vocational, and special education	15 615	576	0.79	0.71–0.87		0.81	0.72–0.90		0.93	0.83–1.05		-41.8	-112.8–29.1
3. Associate professionals (health)	18 653	852	0.99	0.90–1.09		1.01	0.92–1.11		1.17	1.05–1.30		122.6	35.1–210.2
3. Associate professionals (nursery and kindergarten)	35 883	1568	0.96	0.90–1.02		0.95	0.89–1.02		1.10	1.01–1.20		143.7	14.8–272.5
3. Associate professionals (special education and care)	12 758	625	1.03	0.92–1.14		1.02	0.91–1.13		1.17	1.04–1.32		91.5	19.9–163.0
3. Associate professionals, not elsewhere classified	41 749	2076	1.04	0.98–1.10		1.04	0.98–1.10		1.20	1.11–1.29		338.9	192.9–485.0
3. Clerks without customer contacts	44 230	2127	1.03	0.97–1.09		1.03	0.97–1.09		1.18	1.10–1.28		328.6	177.4–479.8
3. Clerks, not elsewhere classified	13 661	735	1.12	1.01–1.24		1.11	1.01–1.22		1.28	1.15–1.43		162.4	87.2–237.5
3. Customer service clerks	17 350	866	1.06	0.96–1.16		1.06	0.97–1.16		1.22	1.10–1.36		159.0	72.8–245.2
3. Nurses and midwives	38 133	1677	0.97	0.91–1.03		0.98	0.92–1.05		1.14	1.04–1.24		200.3	64.8–335.7
3. Primary School teachers	30 583	1192	0.86	0.80–0.93		0.87	0.81–0.94		1.01	0.92–1.11		7.0	-105.1–119.1
3. Public safety workers	2303	111	0.99	0.77–1.28		1.00	0.77–1.29		1.15	0.88–1.50		14.5	-14.7–43.6
3. Sales and service workers, not elsewhere classified	1919	96	1.03	0.79–1.33		1.00	0.77–1.30		1.16	0.89–1.50		12.9	-12.3–38.2
3. Travel attendants and related workers	1707	90	1.06	0.79–1.40		1.06	0.80–1.39		1.22	0.92–1.62		16.2	-9.0–41.4
4. Assembly workers	2183	127	1.28	0.98–1.65		1.23	0.98–1.54		1.42	1.12–1.79		37.4	8.0–66.8
4. Childcare workers in private homes	19 206	855	0.97	0.89–1.06		0.96	0.87–1.04		1.10	1.00–1.22		79.7	-5.8–165.2
4. Cleaners, janitors and kitchen helpers	17 693	895	1.07	0.98–1.17		1.06	0.97–1.16		1.22	1.11–1.35		163.7	78.1–249.2
4. Cooks and housekeepers	7894	412	1.10	0.96–1.26		1.08	0.95–1.22		1.24	1.08–1.42		80.1	24.7–135.6
4. Drivers and manual workers in construction or manufacturing industries	6015	320	1.11	0.96–1.29		1.09	0.94–1.25		1.25	1.08–1.46		64.7	17.0–112.5
4. Food and beverage production workers	4432	237	1.14	0.95–1.36		1.10	0.93–1.30		1.27	1.06–1.51		50.0	8.8–91.3
4. Hairdressers, beauticians, and related workers	7358	363	1.03	0.89–1.19		1.02	0.89–1.18		1.18	1.02–1.37		55.5	2.2–108.9
4. Home care workers	26 603	1496	1.23	1.14–1.33		1.17	1.10–1.25		1.35	1.24–1.47		388.7	273.3–504.2
4. Nursing home workers	20 377	1038	1.12	1.02–1.22		1.08	1.00–1.18		1.25	1.13–1.38		207.5	112.6–302.4
4. Painters	2277	98	0.92	0.71–1.19		0.88	0.67–1.15		1.02	0.77–1.34		1.7	-25.1–28.5
4. Production and plant operators	3088	170	1.17	0.95–1.46		1.14	0.93–1.39		1.31	1.07–1.61		40.6	6.3–74.9
4. Shop assistants	29 683	1610	1.10	1.03–1.18		1.08	1.02–1.16		1.25	1.15–1.36		321.7	198.7–444.6
4. Skilled and unskilled agricultural, forest, and fishery workers	4672	231	1.05	0.88–1.25		1.08	0.91–1.28		1.25	1.04–1.49		45.7	4.8–86.7
4. Skilled workers, not elsewhere classified	3622	185	1.07	0.88–1.30		1.06	0.87–1.28		1.22	1.00–1.49		33.4	-2.6–69.4
4. Unskilled workers, not elsewhere classified	1514	67	0.88	0.66–1.18		0.87	0.64–1.18		1.00	0.73–1.37		0.0	-21.0–21.1
4. Unstated	49 154	2500	1.05	1.00–1.11		1.05	1.00–1.11		1.21	1.13–1.30		437.7	272.3–603.1
4. Waiting staff and bartenders	2038	96	0.89	0.70–1.15		0.88	0.68–1.14		1.02	0.78–1.33		1.5	-24.1–27.1

* Adjusted for maternal age, BMI, smoking, parity, marital status, country of origin, calendar period of birth, and quarter of conception, and sex of the child. ^a^ Job group classification by social class is described in Supplementary, section S6. ^b^ Minimally adjusted model with average risk as reference. Adjusted for maternal age, parity, calendar period of birth, and quarter of conception, and sex of the child. ^c^ Adjusted model with average risk as reference. ^d^ Adjusted model with the simulation–based lowest risk as reference.

**Figure 2:**
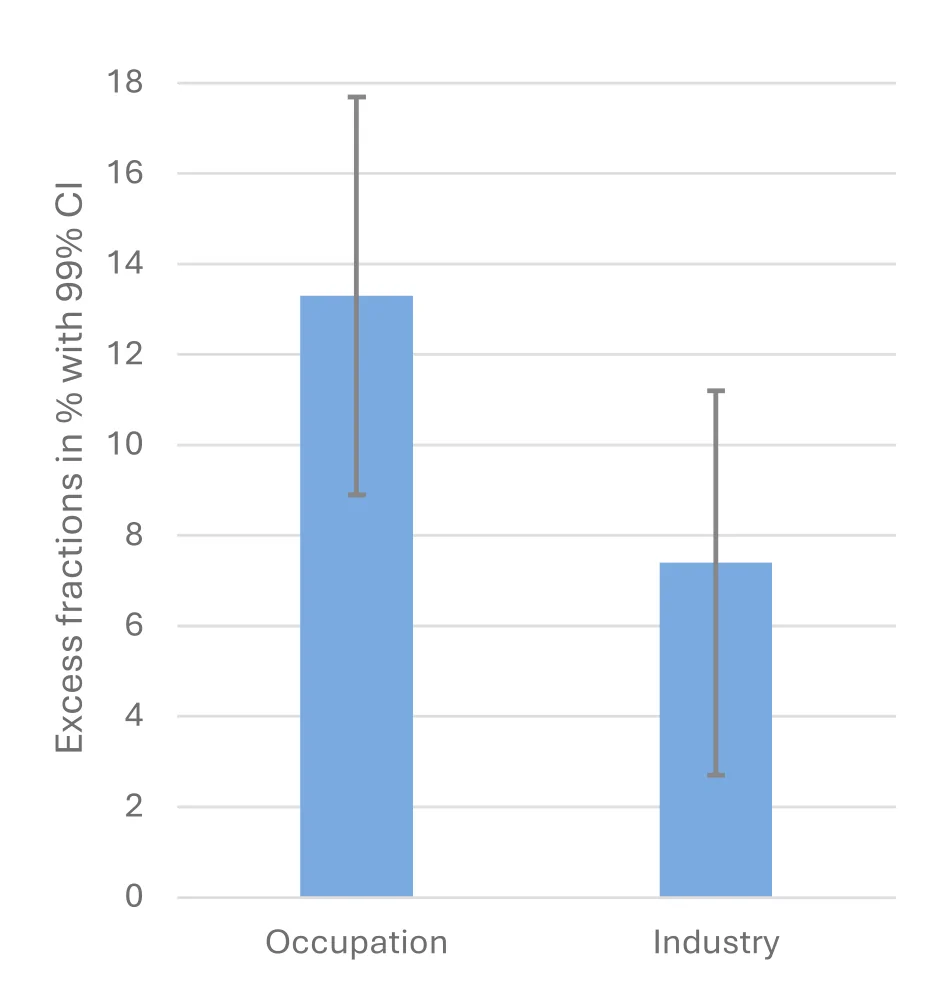
Estimated excess fractions in percentages (bars) and 99% confidence intervals (CI) (black lines) of preterm birth by job and industry, derived from Monte Carlo simulation.

The excess fraction was estimated to be 0.13 (99% CI 0.09–0.18) (figure 2) indicating that approximately 13% of the PTB cases would not have occurred among the studied women if the risk in every occupational group had been equal to that of the simulation-based lowest risk group. As expected, a higher number of occupational groups showed statistically significantly increased risks when compared with the simulation-based lowest risk than with the average risk. Hence, 22 out of 38 occupational groups had a statistically significantly increased risk of PTB. The highest RR was observed for assembly workers (1.42, 99% CI 1.12–1.79), home care workers (1.35, 99% CI 1.24–1.47) and production and plant operators (1.31, 99% CI 1.07–1.61). Because women with unstated occupation and home care workers had both increased risk and a high number of pregnancies, these groups also had the highest estimated numbers of excess cases of PTB during the study period, corresponding to approximately 272–603 excess cases among women with unstated occupation, and 273–504 excess cases among home care workers. For associate professionals (not elsewhere classified), clerks without customer contact, and shop assistants, the number approximated 300 per occupational group, consistent with their increased risks and large number of pregnancies.

### Industry and PTB

Overall, industrial group was associated with PTB (table 3). In the minimally adjusted model 1, several industrial groups showed elevated or reduced risk compared with the overall average. The general pattern was the same in model 2, although fewer industries showed statistically significant differences, and the risk estimates were slightly less pronounced. Results from model 2 showed that pregnancies in manufacture of metals and machinery and residential centers and home help had higher risks for PTB compared to the average risk. In contrast, pregnancies in publishing, broadcasting, and motion pictures and research and university education were less likely to end in PTB. Using the simulation-based lowest risk as reference in model 3, we estimated an excess fraction of 0.07 (99% CI 0.03–0.11) (figure 2). In total, ten industrial groups showed a statistically significantly increased risk of PTB. The highest risks were observed for agriculture, forestry and fishing (RR 1.29, 99% CI 1.06–1.57), manufacture of metals and machinery (RR 1.22, 99% CI 1.07–1.40), and manufacture of electronic components and equipment (RR 1.20, 99% CI 1.01–1.42). The highest excess numbers of PTB during the study period were observed in residential centers and home help (238–670 cases) and among hospital workers (122–494 cases).

**Table 3:** Number of births (N), observed number of cases of preterm birth, risk ratios (RR), and excess cases with 99% confidence interval (CI) by industrial group, ordered alphabetically (total N=644 349). [BMI=body mass index.]

Industrial group	N	Observed cases	Model 1 ^a^			Model 2 ^b,*^			Model 3 ^c,*^			Excess cases ^c,*^	
			RR	99% CI		RR1	99% CI		RR2	99%CI		Cases	99% CI
Agriculture, forestry, and fishing	3206	183	1.18	0.96–1.45		1.19	0.99–1.45		1.29	1.06–1.57		41.1	5.4–76.8
Building completion and finishing	3874	194	1.07	0.88–1.30		1.00	0.82–1.20		1.08	0.89–1.31		13.6	-24.0–51.3
Civil engineering	792	33	0.88	0.58–1.34		0.89	0.57–1.39		0.96	0.61–1.51		-1.3	-16.2–13.5
Cleaning industry	14 108	705	1.07	0.96–1.18		1.01	0.91–1.11		1.09	0.98–1.21		56.8	-17.7–131.3
Construction and demolition of buildings	1854	80	0.92	0.70–1.22		0.93	0.69–1.24		1.00	0.75–1.34		0.1	-23.4–23.7
Culture and sports	8953	412	0.90	0.80–1.02		0.93	0.82–1.06		1.01	0.88–1.15		2.2	-53.9–58.2
Daycare (all ages)	62 721	2834	0.99	0.94–1.04		0.97	0.92–1.02		1.04	0.97–1.12		118.2	-69.7–306.2
Defense, security, and justice activities	7821	345	0.93	0.81–1.07		0.91	0.79–1.05		0.98	0.84–1.14		-6.5	-58.7–45.7
Energy, mining, and quarrying	1683	58	0.74	0.55–1.00		0.76	0.54–1.08		0.82	0.58–1.17		-12.5	-32.9–8.0
Finance and insurance activities	21 232	999	1.00	0.91–1.09		0.97	0.89–1.06		1.05	0.95–1.16		48.9	-47.4–145.2
Hairdressing and other personal service activities	8969	421	0.96	0.84–1.09		0.91	0.79–1.03		0.98	0.85–1.12		-9.5	-67.9–48.8
Health practitioners and veterinarians	23 022	1015	0.97	0.90–1.05		1.04	0.95–1.13		1.12	1.02–1.23		109.3	15.1–203.4
Hospitals	64 354	2845	0.99	0.94–1.04		1.04	0.99–1.09		1.12	1.05–1.20		308.1	122.5–493.8
Hotel and other accommodation facilities	5310	247	0.90	0.77–1.06		0.88	0.75–1.04		0.95	0.80–1.13		-12.6	-55.1–29.9
IT and telecommunications	9798	490	1.04	0.92–1.17		1.06	0.94–1.19		1.15	1.01–1.30		62.1	1.0–123.1
Manufacture and repair of vehicles	1236	61	1.06	0.75–1.49		1.02	0.73–1.43		1.10	0.79–1.54		5.8	-14.7–26.2
Manufacture of chemicals and pharmaceuticals	8712	349	0.86	0.75–0.98		0.90	0.78–1.03		0.97	0.83–1.12		-11.8	-64.4–40.8
Manufacture of electronic components	4734	250	1.13	0.95–1.35		1.11	0.94–1.31		1.20	1.01–1.42		41.8	-0.6–84.3
Manufacture of food products	6619	352	1.11	0.96–1.28		1.08	0.94–1.24		1.16	1.00–1.34		48.8	-2.1–99.7
Manufacture of meat products	2809	147	1.13	0.90–1.43		1.07	0.87–1.33		1.16	0.93–1.44		20.2	-11.9–52.2
Manufacture of metals and machinery	7785	418	1.15	1.00–1.32		1.13	1.00–1.29		1.22	1.07–1.40		76.6	20.8–132.4
Manufacture of plastic, glass, and concrete	4004	192	1.02	0.84–1.23		1.01	0.83–1.22		1.09	0.89–1.32		15.4	-21.8–52.7
Non–university education and training	57 822	2374	0.92	0.87–0.97		0.96	0.91–1.02		1.04	0.96–1.12		87.3	-80.8–255.3
Other private offices and associations	60 364	2792	0.95	0.90–0.99		0.97	0.93–1.02		1.05	0.98–1.12		136.2	-48.5–320.9
Public administration	32 732	1420	0.95	0.89–1.02		0.99	0.92–1.06		1.07	0.98–1.16		91.0	–26.0–208.1
Publishing, Broadcasting, and motion pictures	9929	412	0.83	0.74–0.94		0.85	0.75–0.97		0.92	0.80–1.05		-37.2	-94.6–20.2
Religious institutions and funerals	2123	86	0.86	0.66–1.12		0.91	0.68–1.21		0.98	0.73–1.31		-1.5	-26.5–23.4
Repair and installation of machinery	563	23	0.86	0.52–1.41		0.85	0.50–1.46		0.92	0.54–1.58		-2.0	-14.4–10.5
Research and university education	15 419	604	0.83	0.75–0.91		0.89	0.80–0.99		0.96	0.85–1.08		-25.6	-96.9–45.7
Residential centers and home help	70 033	3645	1.13	1.08–1.18		1.06	1.01–1.10		1.14	1.07–1.22		454.0	238.2–669.8
Restaurants and bars	12 318	633	0.98	0.88–1.08		0.96	0.87–1.07		1.04	0.93–1.16		24.4	-45.9–94.7
Retail trade	52 684	2759	1.05	1.00–1.11		1.02	0.97–1.07		1.10	1.02–1.17		242.3	62.9–421.8
Textile and paper products	3905	191	0.99	0.82–1.20		1.03	0.86–1.25		1.11	0.92–1.35		19.7	-17.1–56.5
Transport of goods	7654	399	1.06	0.93–1.22		1.03	0.90–1.17		1.11	0.97–1.28		40.5	-14.2–95.2
Transport of passengers	3418	159	0.94	0.77–1.14		0.90	0.73–1.10		0.97	0.78–1.20		-5.1	-38.9–28.8
Unstated	10 524	479	0.90	0.80–1.00		0.92	0.82–1.04		1.00	0.88–1.13		-0.7	-60.4–59.0
Water supply, sewage, and waste management	697	40	1.23	0.78–1.94		1.21	0.81–1.81		1.31	0.87–1.96		9.4	-6.9–25.6
Wholesale trade	27 991	1383	1.03	0.96–1.11		1.03	0.96–1.11		1.11	1.02–1.21		140.1	27.0–253.2
Wood products and furniture	2577	138	1.15	0.90–1.46		1.13	0.90–1.41		1.22	0.97–1.53		24.6	-6.5–55.7

*Adjusted for maternal age, BMI, smoking, parity, marital status, country of origin, calendar period of birth, quarter of conception, socioeconomic status, and sex of the child. ^a^ Minimally adjusted model with average risk as reference. Adjusted for maternal age, parity, calendar period of birth, and quarter of conception, and sex of the child. ^b^ Adjusted model with average risk as reference. ^c^ Adjusted model with the simulation–based lowest risk as reference.

### Sensitivity analyses

In sensitivity analyses, model 2 was applied only. First, PTB was subcategorized as very preterm and moderately preterm. For very PTB, the only occupational group with statistically significantly increased risk was home care workers (supplementary table S5). For industries, residential centers and home help remained with statistically significantly increased risk, and transport of goods also showed elevated risk (supplementary table S6). For moderately PTB, the results for occupation were consistent with the main model 2, while for industries, residential centers and home help and agriculture, forestry and fishing had statistically significant increased risks. Analyses restricted to first-born children during the study period and with further adjustment for household income yielded results consistent with the main analyses (supplementary table S7–S10). In time-stratified analyses, two occupational groups showed lower risk during 2004–2011 and five during 2012–2018 (supplementary table S11–S12); home care workers and women with unstated occupation had statistically significantly increased risks in the early period (supplementary table S11), while home care workers and shop assistants had increased risks in the later period (supplementary table S12). For industries, residential centers and home help was the only group to show a statistically significantly increased risk during the early period (supplementary table S13–S14), with no statistically significant differences during the later period. Analyses restricted to women with Denmark as the country of origin were consistent with the main findings for occupational group (supplementary table S15). For industries, the agriculture, forestry, and fishing sector showed a statistically significant increased risk (RR 1.30, 99% CI 1.01–1.68), in addition to the industrial groups already identified in the main analysis (supplementarytable S16).

## Discussion

### Summary of results

In this nationwide register-based study of employed women giving birth to 644 349 live-born singletons in Denmark, 2004–2018, 4.7% of the pregnancies ended in PTB. The risk of PTB varied across maternal occupational and industrial groups. Among occupational groups, 5 of the 38 groups had statistically significantly increased risk of PTB compared with the average population risk, including home care workers, clerks (not elsewhere classified), nursing home workers, shop assistants, and workers with unstated job codes. Compared to the simulation-based lowest risk reference, 22 groups had increased risk, with assembly workers, home care workers, and production and plant operators showing the highest RR. As home care workers and nursing home workers represented large groups of pregnant employees, comparison with the simulation-based lowest risk indicated that as many as 273–504 and 112–302 cases of PTB, respectively, could potentially have been prevented during the study period. The industry analyses showed statistically significantly higher risks for 2 of the 39 industrial groups compared with the average risk and for 10 groups compared with the simulation-based lowest risk. The highest RR were observed among agriculture, forestry and fishing, and manufacture of metals and machinery, while the highest excess numbers of PTB during the study period were observed for residential centers and home help and hospital workers, two industries with many pregnant employees during the study period.

### Statistical methodological discussion

Before interpreting the results, it is important to consider the statistical approach used to estimate excess fractions and RR using the simulation-based lowest risk as the reference (model 3). This method was included to explore the preventive potential across occupational and industrial groups, as comparisons with the average population risk (model 2) do not provide this perspective. The approach allows estimation of excess fractions when no natural reference group exists, data are structured at group level, and individual observations are correlated. We used a Monte Carlo simulation to create a synthetic lowest-risk reference group and estimate the proportion of PTB theoretically attributable to occupational or industrial group. An important strength is that it accounts for random variation and eliminates the need to identify a single group with the absolute lowest risk. Similar procedures have been used in other studies lacking natural reference groups, typically through population-attributable risk approaches based on independent observations (30–34). In contrast, our analysis accounted for correlated individual-level data (20 (Hannerz H, Andersen AJ. A simulation-based method for estimating excess fractions without a natural reference group. Biostat Epidemiol. Submitted)).

After estimating the excess fraction, we calculated model 3 RR using the simulation-based lowest risk as synthetic reference group (20). In the following discussion, we refer primarily to these RR as they better reflect differences across occupational and industrial groups than comparisons with the population average.

### Strengths and limitations

A key strength of this study was the ability of linking various nationwide registry data at the individual level. This enabled us to include nearly all Danish women employed during pregnancy over a 14-year period and reduced selection bias. The detailed information allowed us to assign each pregnancy to major occupational and industrial groups and to adjust for important potential confounding factors such as maternal country of origin, age, smoking during pregnancy, and pre-pregnancy BMI (35), and with few missing (0–3%). The large study population enabled us to study PTB, a relatively rare outcome, across a wide range of 38 occupational and 39 industrial groups.

Some limitations should be noted. Misclassification of occupation or industry is possible, as information was based on the woman’s main income source in the calendar year of conception. Women who changed employment before or during pregnancy may therefore have been classified under a previous job. Such misclassification could be differential if women in certain jobs were more likely to change employment after becoming pregnant, although changes likely would have occurred within similar occupational or industrial groups. Because employment was classified according to the main source of income in the calendar year of conception, the actual proportion of pregnancy spent in that occupation may differ depending on when conception occurred within the year. This was addressed by adjusting for quarter of conception. Misclassification of gestational age is possible as the Danish Medical Birth Registry relies on clinical reporting (23), although ultrasound screening has been used for >90% of all pregnancies since the introduction of nationwide ultrasound screening in 2004 (36). PTB constitutes a heterogeneous outcome encompassing different clinical entities (eg, spontaneous versus medically indicated birth, early versus late PTB) with potentially different etiologies (17). We used a binary outcome for PTB, which may increase sensitivity to misclassification. However, registry validity of the medical birth register is high (23), misclassification is expected to be non-differential, and sensitivity analyses including additional preterm categories supported findings from the main analysis (supplementart table S5–S6). Prior research has shown that hazardous environmental exposures cause live-birth bias, especially among socially vulnerable women (37). Restricting the study to live births may introduce selection bias (38), potentially underestimating effects if pregnancy loss occurs more frequently among groups at higher risk of PTB. Exclusion of multiple pregnancies may have reduced the overall prevalence of PTB, as multiple pregnancies are more likely to end preterm (39). Companies with <10 employees are not required to report DISCO codes. This may limit generalizability of results to small enterprises and may possibly lead to underestimation of risks if working conditions are poorer in such settings. As the DOC*X Generation cohort terminated at the end of 2018 (21), generalizability of the results to later or more recent periods may be limited. However, by not including these periods, we avoided the variation in work conditions inferred by COVID-19. Finally, the Danish study population was relatively homogeneous, well-resourced, with relatively high levels of education and free access to prenatal health care. On the one hand this reduces the potential for residual confounding related to SES, health related behavior and access to health care. On the other hand, it – together with the generally low risk of PTB – may reduce generalizability to populations with more diversity in education, health and resources. In countries with less stringent regulation and control of occupational hazards in relation to pregnancy than in the Nordic countries, occupational risk factors may have a much stronger impact on the occurrence of PTB.

### Interpretation of results

We aimed to identify groups with increased risk among pregnant Danish workers to provide evidence-based guidance for further studies and preventive actions, without focusing on specific risk factors. It is however relevant to consider our findings in the light of previous literature to understand possible underlying explanations for our findings.

A broad range of common factors in the work environment have been associated with increased risk of PTB, including physical workload, psychosocial work conditions, and exposure to some chemicals (7–15, 40). Consistent with these findings, we observed the highest and most consistent risks among care workers (home care workers, nursing home workers, and employees in the residential centers and home help industry). These occupational groups typically involve both high physical and psychosocial workloads and shift work in Denmark (41). Although home care workers and nursing home workers have similar tasks, the risk was higher among home care workers, possibly because their work takes place in private homes, where adaption of job tasks due to pregnancy are limited and exposure to tobacco smoke, cleaning chemicals, infections and inadequate assistive help and equipment are frequent.

However, also other occupational groups (ie, clerks (not elsewhere classified), shop assistants, and employees in the manufacture of metals and machinery industry) showed statistically significantly increased risks. Previous evidence for these specific groups is scarce, but for the latter group, metal exposure might play a role as, for example, exposure to iron, welding fumes and heavy metals have been associated with PTB (42, 43). Previous studies on the association between job strain and adverse pregnancy outcomes found that high job strain (high psychological demands and low job control) was associated with higher incidence of PTB (9, 10). This could be expected to particularly influence work as shop assistants, who in combination with a lot of standing and walking at work, may also have many contacts and limited control over their work. The same could apply to clerks.

In the same cohort as ours, Begtrup et al (26) identified occupational and industrial groups with high pregnancy-related absence. To a large extent, these groups overlap with those at increased risk of PTB. It could be argued that work absence would have prevented exposure to occupational hazards and therefore also risk of PTB. However, absence data did not distinguish between leave due to unsafe work conditions and pregnancy complications, which themselves may increase the risk of PTB (44). Importantly, exposures very early in pregnancy, and therefore prior to most pregnancy-related absence, may be critical for risk of PTB (17). Interestingly, an earlier Danish study found absence from work to increase with the number of risk factors experienced at work (45). The occupational groups with increased risks are generally characterized by exposure to several different risk factors for preterm birth. In future studies, it might be important to address whether it is the sum of exposure to risk factors, at work and in private life, that opens the path to preterm birth rather than individual factors (45, 46).

We found that the large group of employees with unstated job code had increased risk of PTB in all three statistical models. The 272–603 excess cases of PTB during the study period represent a large potential for prevention. A major part of this group may include workers in small businesses with <10 employees, for which the employer is not obliged to register a DISCO-code. It is likely that such businesses do not have the same level of preventive occupational health and safety measures for pregnant women as larger companies.

Socioeconomically disadvantaged groups have higher incidence of PTB, even with universal access to healthcare (47, 48). This should be acknowledged when interpreting our findings, as occupation is strongly associated with social class and correlates with several established risk factors for PTB (49). We adjusted for several important confounders, nonetheless we did not have access to data on other potentially relevant factors, including chronic diseases such as diabetes, obstetric complications, alcohol use, diet, and healthcare utilization. Because such factors may be associated with both socioeconomic position and preterm birth, we cannot rule out residual confounding from unmeasured variables. However, the sensitivity analysis with additional adjustment for household income yielded results consistent with the main analyses. When the results from the occupational group analyses are viewed according to classification by social class (c.f. supplementary material S6), several working-class groups do not show elevated risk. Thus, only 4 of the 16 occupational groups in the lowest social class show significantly increased risk compared to the population average (table 2). Further, although high pre-pregnancy BMI and smoking during pregnancy were common among the occupational groups with statistically significantly increased risk of PTB compared to the population average, other occupational groups with high prevalence of smoking and high BMI did not show such increased risks (eg, cleaners, janitors and kitchen helpers, cooks and housekeepers, and painters). Overall, this indicates that occupational exposures contribute to risk, but also that there may be an interplay between work-related and lifestyle factors (35). It is therefore noteworthy that a previous study reported stronger effects of shift work among low-income workers (50), which may explain why medical doctors, who also work shifts, showed lower risk compared with the average in our study.

As the analyses were based on groups, the increased risks could not be linked to specific work conditions. The results should therefore not be interpreted as evidence that all jobs within a given group are hazardous, but the elevated risks observed in some occupational and industrial groups do suggest that existing measures may not be sufficient or sufficiently implemented in all work settings. Greater attention could be given to early workplace risk assessment during pregnancy, or, preferably, formulation of workplace policies for pregnant workers, enabling the employer to modify work tasks immediately upon announcement of the pregnancy. Further, awareness of potential risk factors may encourage the pregnant employees to announce their pregnancy early to benefit from modification of work tasks, and hence, reduced risks for their unborn child.

### Concluding remarks

This nationwide register-based study estimated the risk of PTB across a broad range of maternal occupational and industrial groups. Several groups showed elevated risks, particularly home care workers, suggesting that the risk of PTB differs across maternal occupational and industrial groups. Overall, our results contribute to a broader understanding of how factors in the work environment may contribute to PTB and support consideration of occupational exposures in maternal health research and policy.

## Supplementary material

Supplementary material
