# Supplementary material for: Relative risk and excess fraction of preterm birth across maternal occupation and industry: a Danish nationwide register-based cohort study of employed pregnant women
Source: Scand J Work Environ Health. 2026 Aug 31;52(5):502–13. doi: 10.5271/sjweh.4310 (PMC13542837; doi:10.5271/sjweh.4310)
Supplement: Supplementary file 1 [file SJWEH-52-502-S001.pdf]

**Relative risk and excess fraction of preterm birth across maternal occupation and industry: a Danish nationwide register-based cohort study of employed pregnant women**[1]

***by Astrid Juhl Andersen, PhD,[2] Marie Pedersen, PhD, Harald Hannerz, PhD, Sandra Søgaard Tøttenborg, PhD, Esben Meulengracht Flachs, PhD, Jens Peter Ellekilde Bonde, DSc, Jenny Selander, PhD, Luise Mølenberg Begtrup, PhD, Camilla Sandal Sejbaek, PhD, Karin Sørig Hougaard, PhD***

1. Supplementary materials
2. Correspondence to: Astrid Juhl Andersen, The National Research Centre for the Working Environment, Lersø Parkallé 105, 2100 København Ø, Denmark. [E-mail: aja@nfa.dk]

## Overview

S1: Deviations from the published study protocol .......... 2
S2: Overview over models, adjustment and reference group (Figure S1 and S2) .......... 3
S3: Sociodemographic characteristics of occupational and industrial groups (Table S1-S2) .......... 4
S4: Results from the minimally adjusted analyses (Table S3-S4) .......... 7
S5: Sensitivity analyses .......... 9
Subdivided outcome of preterm birth intro three categories (Table S5-S6) .......... 9
Restriction to firstborns (Table S7-S8) .......... 14
Further adjustment for household income (Table S9-S10) .......... 16
Analyses stratified on time (Table S11-S14) .......... 18
Analyses restricted to mothers with Denmark as country of origin (Table S15-S16) .......... 22
S6: Job group classification by social class .......... 24

# S1: Deviations from the published study protocol

1. Due to the high number of rate ratios reported, we decided to use 99% Confidence Intervals (CI), instead of 95% CIs. However, a 95% CI was mistakenly stated in two of three columns in the protocol's Table 3.

2. We have added two more industrial groups. After the publication of the study protocol, we became aware of the advantage of grouping the industrial group "17: Administration, brokerage, consultancy, financing and insurance activities" into three groups: "17.1: Public administration", "17.2: Finance and insurance activities" and "17.3: Other private offices and associations". We therefore include a total of 38 industrial groups. This grouping has previously been used by the Danish Working Environment Authority.

3. The total number of individuals included in our analyses (N=644,349) is higher than we initially presented in the flow chart in the study protocol (N=632,886). When we prepared the flow chart for the study protocol, we excluded those who were "independent". These have been included in the analyses, which is consistent with what we state in the study protocol (under inclusion criteria).

4. Specification of the results table presented in the study protocol: We present risks rates (RR) from the minimal adjusted model (stated in the protocol: "Crude RR"). Further, all tables on occupational groups were organized according to social class (c.f. the job group classification in S6), with job groups within each social class ordered alphabetically.

5. Sensitivity analyses:
   a. We planned to run analyses on four outcome categories of preterm birth. However, since there were too few cases of extremely preterm birth in some of the occupational and industrial groups, we grouped "Extremely preterm birth" and "Very preterm birth" together, resulting in three outcome categories.
   b. We added more sensitivity analyses than initially planned, including:
      i. Further adjustment of household income in an extra model.
      ii. Time stratified analyses: Divided observations into two periods.
      iii. Analyses restricted to mothers with Denmark as country of origin.
      iv. Minimal adjusted model.

# S2: Overview over models, adjustment and reference group (Figure S1 and S2)

Figure S1: Directed acyclic graph for identification of confounders

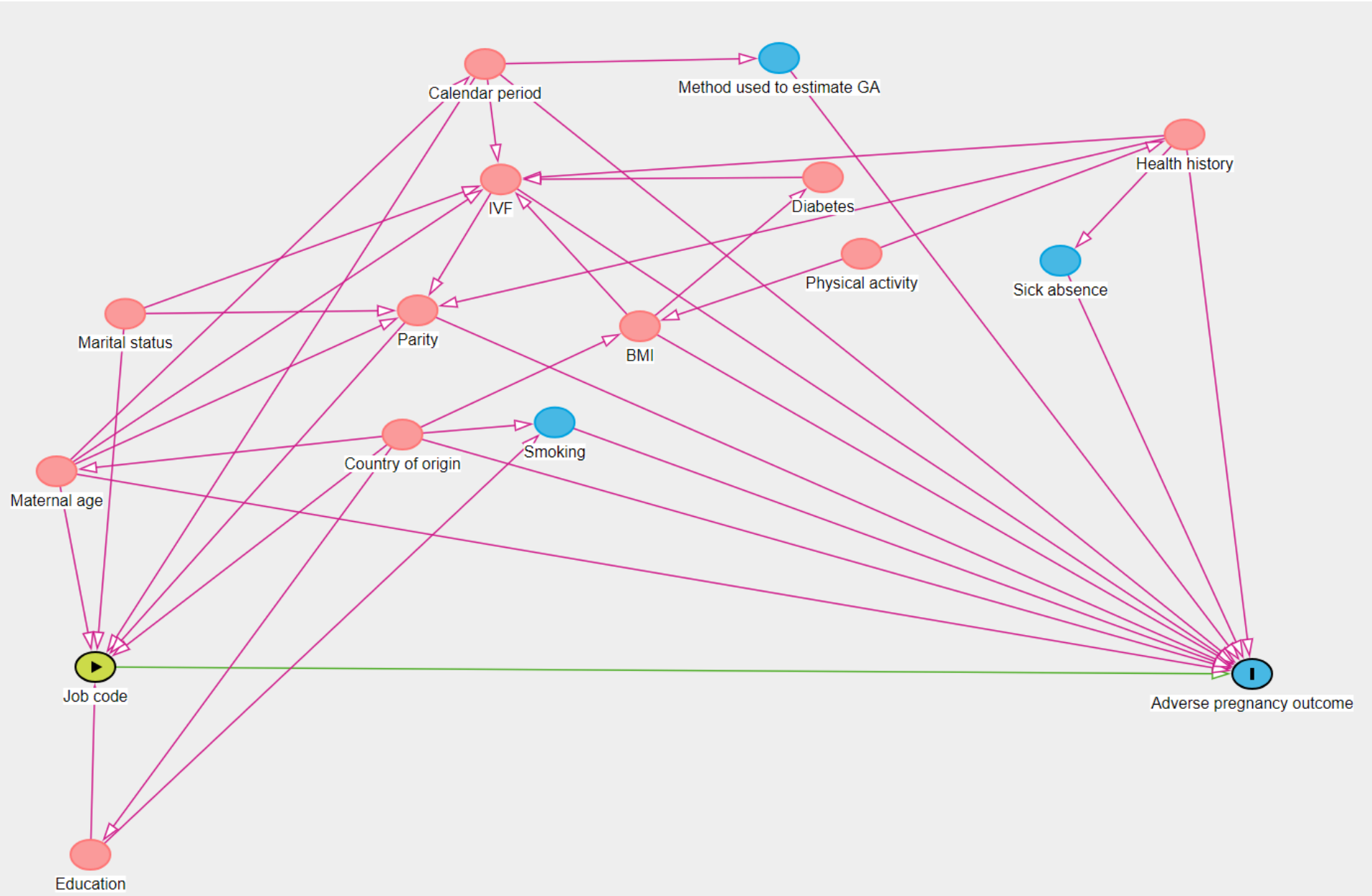


Figure S2: overview of the three statistical models

| **Model** | **Adjustment level** | **Comparison group** |
|---|---|---|
| Model 1 | Minimal | Average risk in total study population |
| Model 2 | Full | Average risk in total study population |
| Model 3 | Full | Simulation-based lowest-risk |

# S3: Sociodemographic characteristics of occupational and industrial groups (Table S1-S2)

Table S1: Sociodemographic characteristics of the occupational groups, ordered by social class (1. to 4.) and alphabetically within social class. Number of births (N), preterm birth cases, median age, and percentage of the observations in the occupational group belonging to the highest risk category for the mother's country of origin, BMI and smoking.

| **Occupational group and social class** | **N** | **Preterm birth cases (%)** | **Age, median [IQR]** | **Origin from outside of Europe or unstated, %** | **BMI ≥30, %** | **Smoking during pregnancy, %** |
|---|---|---|---|---|---|---|
| 1.Medical doctors, dentists, and veterinarians | 12 537 | 435 (3.8) | 31.8 (29.6--34.6) | 5.0 | 2.8 | 0.7 |
| 1.Professionals (physics, mathematics, engineering, architects) | 15 350 | 613 (4.0) | 32.4 (30.0--35.2) | 6.0 | 5.9 | 2.4 |
| 1.Professionals at academic level, not elsewhere classified | 54 556 | 2229 (4.1) | 32.3 (29.8--35.1) | 2.5 | 5.1 | 2.6 |
| 1.Senior officials and corporate managers | 6035 | 285 (4.7) | 33.4 (30.4--36.5) | 2.3 | 8.3 | 6.5 |
| 2.Associate professionals (business and administration) | 51 144 | 2325 (4.6) | 31.6 (28.9--34.6) | 2.5 | 7.9 | 5.7 |
| 2.Associate professionals (physics and engineering) | 10 065 | 433 (4.3) | 31.7 (29.0--34.8) | 3.9 | 10.1 | 5.6 |
| 2.Managers (<10 employees) | 2247 | 112 (5.0) | 30.9 (27.7--34.3) | 5.3 | 13.0 | 10.8 |
| 2.Psychologists and social workers | 10 062 | 444 (4.4) | 31.5 (29.0--34.4) | 2.8 | 8.2 | 4.4 |
| 2.Teachers in higher, secondary, vocational, and special education | 15 615 | 576 (3.7) | 32.3 (29.8--35.2) | 4.2 | 5.6 | 2.2 |
| 3. Associate professionals (health) | 18 653 | 852 (4.6) | 30.3 (27.9--33.1) | 3.0 | 7.0 | 3.8 |
| 3.Associate professionals (nursery and kindergarten) | 35 883 | 1568 (4.4) | 30.4 (27.8--33.4) | 1.6 | 13.8 | 7.4 |
| 3.Associate professionals (special education and care) | 12 758 | 625 (4.9) | 30.3 (27.5--33.3) | 0.7 | 15.8 | 10.6 |
| 3.Associate professionals, not elsewhere classified | 41 749 | 2076 (5.0) | 28.5 (25.2--32.1) | 7.5 | 12.7 | 13.6 |
| 3.Clerks without customer contacts | 44 230 | 2127 (4.8) | 30.5 (27.6--33.7) | 2.9 | 12.4 | 8.4 |
| 3.Clerks, not elsewhere classified | 13 661 | 735 (5.4) | 29.8 (26.8--33.1) | 3.1 | 11.7 | 12.3 |
| 3.Customer service clerks | 17 350 | 866 (5.0) | 30.4 (27.7--33.3) | 2.9 | 9.7 | 6.4 |
| 3.Nurses and midwives | 38 133 | 1677 (4.4) | 30.3 (27.8--33.2) | 1.8 | 9.2 | 4.1 |
| 3.Primary School teachers | 30 583 | 1192 (3.9) | 31.1 (28.6--34.0) | 1.4 | 10.6 | 5.0 |
| 3.Public safety workers | 2303 | 111 (4.8) | 31.3 (28.8--34.1) | 0.6 | 5.5 | 9.8 |
| 3.Sales and service workers, not elsewhere classified | 1919 | 96 (5.0) | 28.9 (25.3--33.0) | 10.7 | 11.6 | 20.0 |
| 3.Travel attendants and related workers | 1707 | 90 (5.3) | 31.6 (28.4--35.0) | 6.6 | 5.4 | 9.9 |
| 4.Assembly workers | 2183 | 127 (5.8) | 29.9 (26.6--33.5) | 8.1 | 20.0 | 24.7 |
| 4.Childcare workers in private homes | 19 206 | 855 (4.5) | 28.3 (24.9--32.1) | 6.3 | 16.4 | 15.0 |
| 4.Cleaners, janitors and kitchen helpers | 17 693 | 895 (5.1) | 29.4 (26.0--33.2) | 24.9 | 14.0 | 19.0 |
| 4.Cooks and housekeepers | 7894 | 412 (5.2) | 28.9 (25.6--32.5) | 8.7 | 18.8 | 15.5 |
| 4.Drivers and manual workers in construction or manufacturing industries | 6015 | 320 (5.3) | 29.0 (25.7--32.6) | 8.2 | 15.7 | 21.5 |
| 4.Food and beverage production workers | 4432 | 237 (5.4) | 28.7 (25.5--32.0) | 6.9 | 19.1 | 23.6 |
| 4.Hairdressers, beauticians, and related workers | 7358 | 363 (4.9) | 28.4 (25.7--31.7) | 2.5 | 9.0 | 13.1 |
| 4.Home care workers | 26 603 | 1496 (5.6) | 27.5 (24.2--31.5) | 10.1 | 21.0 | 26.7 |
| 4.Nursing home workers | 20 377 | 1038 (5.1) | 28.8 (25.8--32.4) | 6.5 | 19.0 | 18.7 |
| 4.Painters | 2277 | 98 (4.3) | 27.7 (24.9--31.0) | 0.2 | 13.0 | 25.0 |
| 4.Production and plant operators | 3088 | 170 (5.5) | 30.1 (26.8--33.5) | 6.1 | 18.4 | 23.9 |
| 4.Shop assistants | 29 683 | 1610 (5.4) | 27.6 (24.5--31.1) | 4.2 | 13.9 | 15.7 |
| 4.Skilled and unskilled agricultural, forest, and fishery workers | 4672 | 231 (4.9) | 28.8 (25.8--32.1) | 2.7 | 12.0 | 11.5 |
| 4.Skilled workers, not elsewhere classified | 3622 | 185 (5.1) | 30.7 (27.5--34.0) | 3.2 | 13.6 | 14.7 |
| 4.Unskilled workers NEC | 1514 | 67 (4.4) | 28.8 (25.5--32.5) | 11.0 | 10.2 | 17.0 |

| | | | | | | |
|---|---|---|---|---|---|---|
| 4.Unstated | 49 154 | 2500 (5.1) | 30.0 (26.6--33.5) | 8.4 | 10.3 | 13.6 |
| 4.Waiting staff and bartenders | 2038 | 96 (4.7) | 27.6 (24.8--30.9) | 4.8 | 6.6 | 21.3 |
| NEC=Not elsewhere classified. Job group classification by social class is described in Supplementary, section S6. | | | | | | |

Table S2: Sociodemographic characteristics in industrial groups, ordered alphabetically. Number of pregnancies (N), preterm birth cases, median age, and percentage of observations in the industrial group belonging to the highest risk category for the mother's country of origin, BMI and smoking.

| **Industrial group** | **N** | **Preterm birth cases (%)** | **Age, median [IQR]** | **Origin from outside of Europe or unstated, %** | **BMI ≥30, %** | **Smoking during pregnancy, %** | **Low SES, %** |
|---|---|---|---|---|---|---|---|
| Agriculture, forestry and fishing | 3206 | 183 (5.7) | 28.1 (25.4-31.2) | 1.4 | 10.9 | 11.9 | 67.2 |
| Building completion and finishing | 3874 | 194 (5.0) | 28.6 (25.3-32.1) | 1.2 | 14.1 | 21.0 | 69.4 |
| Civil engineering | 792 | 33 (4.2) | 30.8 (27.5-34.1) | 1.6 | 11.0 | 9.0 | 15.8 |
| Cleaning industry | 14 108 | 705 (5.0) | 29.3 (25.9-33.1) | 25.9 | 13.5 | 18.4 | 89.6 |
| Construction and demolition of buildings | 1854 | 80 (4.3) | 30.4 (27.1-33.7) | 3.3 | 11.4 | 11.9 | 21.6 |
| Culture and sports | 8953 | 412 (4.6) | 31.1 (27.8-34.4) | 3.1 | 6.8 | 7.0 | 20.5 |
| Daycare (all ages) | 62 721 | 2834 (4.5) | 30.0 (27.0-33.2) | 3.7 | 14.2 | 9.8 | 30.9 |
| Defense, security and justice activities | 7821 | 345 (4.4) | 31.0 (28.1-34.0) | 1.4 | 9.4 | 9.7 | 33.3 |
| Energy, mining and quarrying | 1683 | 58 (3.5) | 31.8 (29.0-34.5) | 2.5 | 9.5 | 4.6 | 11.0 |
| Finance and insurance activities | 21 232 | 999 (4.7) | 30.9 (28.2-34.0) | 3.1 | 7.6 | 4.3 | 47.2 |
| Hairdressing and other personal service activities | 8969 | 421 (4.7) | 28.5 (25.7-31.9) | 4.0 | 8.8 | 12.9 | 85.6 |
| Health practitioners and veterinarians | 23 022 | 1015 (4.4) | 30.9 (28.1-34.0) | 4.2 | 7.4 | 5.1 | 6.3 |
| Hospitals | 64 354 | 2845 (4.4) | 30.6 (28.1-33.5) | 3.2 | 8.9 | 4.5 | 12.4 |
| Hotel and other accommodation facilities | 5310 | 247 (4.7) | 29.1 (26.0-32.6) | 9.9 | 11.3 | 15.8 | 65.6 |
| IT and telecommunications | 9798 | 490 (5.0) | 31.7 (29.0-34.8) | 6.3 | 8.9 | 6.7 | 15.7 |
| Manufacture and repair of vehicles | 1236 | 61 (4.9) | 30.0 (27.1-33.4) | 4.8 | 16.7 | 16.3 | 41.3 |
| Manufacture of chemicals and pharmaceuticals | 8712 | 349 (4.0) | 32.4 (29.8-35.3) | 5.1 | 6.9 | 4.1 | 9.6 |
| Manufacture of electronic components/equipment | 4734 | 250 (5.3) | 31.3 (28.4-34.6) | 6.6 | 12.1 | 12.6 | 39.2 |
| Manufacture of Food products | 6619 | 352 (5.3) | 30.0 (26.5-33.4) | 4.3 | 13.8 | 15.3 | 51.6 |
| Manufacture of meat products | 2809 | 147 (5.2) | 29.4 (26.2-32.7) | 13.7 | 17.3 | 20.5 | 78.4 |
| Manufacture of metals and machinery | 7785 | 418 (5.4) | 31.1 (28.1-34.3) | 4.3 | 14.3 | 13.2 | 32.6 |
| Manufacture of plastic, glass and concrete | 4004 | 192 (4.8) | 31.4 (28.2-34.5) | 4.1 | 13.3 | 13.1 | 34.6 |
| Non-university education and training | 57 822 | 2374 (4.1) | 31.4 (28.7-34.4) | 2.5 | 10.4 | 5.9 | 7.2 |
| Other private offices and associations | 60 364 | 2792 (4.6) | 31.1 (28.2-34.2) | 4.0 | 8.0 | 7.0 | 13.0 |
| Public administration | 32 732 | 1420 (4.3) | 31.3 (28.6-34.3) | 3.5 | 10.2 | 6.0 | 3.0 |
| Publishing, Broadcasting and motion pictures | 9929 | 412 (4.2) | 32.1 (29.4-35.1) | 2.3 | 5.7 | 6.4 | 11.1 |
| Religious institutions and funeral activities | 2123 | 86 (4.1) | 32.5 (29.2-35.6) | 1.6 | 9.8 | 4.0 | 8.2 |
| Repair and installation of machinery and equipment | 563 | 23 (4.1) | 31.1 (27.9-34.1) | 6.2 | 13.9 | 14.0 | 27.4 |
| Research and university education | 15 419 | 604 (3.9) | 31.7 (29.2-34.6) | 6.3 | 5.3 | 2.7 | 2.3 |
| Residential centers and home care workers | 70 033 | 3645 (5.2) | 28.9 (25.6-32.5) | 6.3 | 18.2 | 19.1 | 66.2 |
| Restaurants and bars | 12 318 | 633 (5.1) | 27.7 (24.3-31.5) | 14.8 | 11.4 | 20.8 | 51.9 |

| | | | | | | | |
|---|---|---|---|---|---|---|---|
| Retail trade | 52 684 | 2759 (5.2) | 28.1 (24.9-31.6) | 4.5 | 13.0 | 14.5 | 57.4 |
| Textile and paper products | 3905 | 191 (4.9) | 29.6 (26.4-33.1) | 2.2 | 10.9 | 11.2 | 28.3 |
| Transport of gods | 7654 | 399 (5.2) | 30.5 (27.3-33.8) | 8.2 | 12.7 | 13.5 | 48.4 |
| Transport of passengers | 3418 | 159 (4.7) | 31.4 (28.2-34.9) | 4.0 | 12.5 | 13.6 | 57.2 |
| Unstated | 10 524 | 479 (4.6) | 29.6 (26.2-33.2) | 9.0 | 11.1 | 15.1 | 24.5 |
| Water supply, sewage and waste management | 697 | 40 (5.7) | 30.8 (27.4-34.2) | 2.3 | 15.1 | 12.8 | 29.1 |
| Wholesale trade | 27 991 | 1383 (4.9) | 31.3 (28.4-34.3) | 3.6 | 8.9 | 8.0 | 20.7 |
| Wood products and furniture | 2577 | 138 (5.4) | 30.2 (27.3-33.6) | 1.7 | 13.3 | 13.3 | 47.3 |

# S4: Results from the minimally adjusted analyses (Table S3-S4)

Table S3: Minimally adjusted model, excluding adjustment for sociodemographic characteristics, with occupational groups ordered by social class (1. to 4.) and alphabetically within social class. Number of births (N), observed number of cases of preterm birth, and adjusted risk ratios (RR) with 99% confidence interval (CI).
N=644 349.

| | | | Compared with the average risk | |
|---|---|---|---|---|
| **Occupational group and social class** | **N** | **Observed cases** | **RR[a]** | **99% CI** |
| 1.Medical doctors, dentists, and veterinarians | 12 537 | 435 | 0.76 | 0.68-0.85 |
| 1.Professionals (physics, mathematics, engineering, architects) | 15 350 | 613 | 0.83 | 0.76-0.92 |
| 1.Professionals at academic level, not elsewhere classified | 54 556 | 2229 | 0.85 | 0.81-0.90 |
| 1.Senior officials and corporate managers | 6035 | 285 | 0.98 | 0.84-1.14 |
| 2.Associate professionals (business and administration) | 51 144 | 2325 | 0.95 | 0.90-1.00 |
| 2.Associate professionals (physics and engineering) | 10 065 | 433 | 0.91 | 0.81-1.02 |
| 2.Managers (<10 employees) | 2247 | 112 | 1.06 | 0.83-1.37 |
| 2.Psychologists and social workers | 10 062 | 444 | 0.96 | 0.85-1.08 |
| 2.Teachers in higher, secondary, vocational, and special education | 15 615 | 576 | 0.79 | 0.71-0.87 |
| 3. Associate professionals (health) | 18 653 | 852 | 0.99 | 0.90-1.09 |
| 3.Associate professionals (nursery and kindergarten) | 35 883 | 1568 | 0.96 | 0.90-1.02 |
| 3.Associate professionals (special education and care) | 12 758 | 625 | 1.03 | 0.92-1.14 |
| 3.Associate professionals, not elsewhere classified | 41 749 | 2076 | 1.04 | 0.98-1.10 |
| 3.Clerks without customer contacts | 44 230 | 2127 | 1.03 | 0.97-1.09 |
| 3.Clerks, not elsewhere classified | 13 661 | 735 | 1.12 | 1.01-1.24 |
| 3.Customer service clerks | 17 350 | 866 | 1.06 | 0.96-1.16 |
| 3.Nurses and midwives | 38 133 | 1677 | 0.97 | 0.91-1.03 |
| 3.Primary School teachers | 30 583 | 1192 | 0.86 | 0.80-0.93 |
| 3.Public safety workers | 2303 | 111 | 0.99 | 0.77-1.28 |
| 3.Sales and service workers, not elsewhere classified | 1919 | 96 | 1.03 | 0.79-1.33 |
| 3.Travel attendants and related workers | 1707 | 90 | 1.06 | 0.79-1.40 |
| 4.Assembly workers | 2183 | 127 | 1.28 | 0.98-1.65 |
| 4.Childcare workers in private homes | 19 206 | 855 | 0.97 | 0.89-1.06 |
| 4.Cleaners, janitors and kitchen helpers | 17 693 | 895 | 1.07 | 0.98-1.17 |
| 4.Cooks and housekeepers | 7894 | 412 | 1.10 | 0.96-1.26 |
| 4.Drivers and manual workers in construction or manufacturing industries | 6015 | 320 | 1.11 | 0.96-1.29 |
| 4.Food and beverage production workers | 4432 | 237 | 1.14 | 0.95-1.36 |
| 4.Hairdressers, beauticians, and related workers | 7358 | 363 | 1.03 | 0.89-1.19 |
| 4.Home care workers | 26 603 | 1496 | 1.23 | 1.14-1.33 |
| 4.Nursing home workers | 20 377 | 1038 | 1.12 | 1.02-1.22 |
| 4.Painters | 2277 | 98 | 0.92 | 0.71-1.19 |
| 4.Production and plant operators | 3088 | 170 | 1.17 | 0.95-1.46 |
| 4.Shop assistants | 29 683 | 1610 | 1.10 | 1.03-1.18 |
| 4.Skilled and unskilled agricultural, forest, and fishery workers | 4672 | 231 | 1.05 | 0.88-1.25 |
| 4.Skilled workers, not elsewhere classified | 3622 | 185 | 1.07 | 0.88-1.30 |
| 4.Unskilled workers NEC | 1514 | 67 | 0.88 | 0.66-1.18 |
| 4.Unstated | 49 154 | 2500 | 1.05 | 1.00-1.11 |
| 4.Waiting staff and bartenders | 2038 | 96 | 0.89 | 0.70-1.15 |

[a] Adjusted for: maternal age, parity, calendar period of birth, and quarter of conception, and sex of the child.
NEC=Not elsewhere classified. Job group classification by social class is described in Supplementary, section S6.

Table S4: Minimally adjusted model, excluding adjustment for sociodemographic characteristics, with industrial groups ordered alphabetically. Number of births (N), observed number of cases of preterm birth, and adjusted risk ratios (RR) with 99% confidence interval (CI). N=644 349.

| | | | Compared with the average risk | |
|---|---|---|---|---|
| **Industrial group** | **N** | **Observed cases** | **RR[a]** | **99% CI** |
| Agriculture, forestry and fishing | 3206 | 183 | 1.18 | 0.96-1.45 |
| Building completion and finishing | 3874 | 194 | 1.07 | 0.88-1.30 |
| Civil engineering | 792 | 33 | 0.88 | 0.58-1.34 |
| Cleaning industry | 14 108 | 705 | 1.07 | 0.96-1.18 |
| Construction and demolition of buildings | 1854 | 80 | 0.92 | 0.70-1.22 |
| Culture and sports | 8953 | 412 | 0.90 | 0.80-1.02 |
| Daycare (all ages) | 62 721 | 2834 | 0.99 | 0.94-1.04 |
| Defense, security and justice activities | 7821 | 345 | 0.93 | 0.81-1.07 |
| Energy, mining and quarrying | 1683 | 58 | 0.74 | 0.55-1.00 |
| Finance and insurance activities | 21 232 | 999 | 1.00 | 0.91-1.09 |
| Hairdressing and other personal service activities | 8969 | 421 | 0.96 | 0.84-1.09 |
| Health practitioners and veterinarians | 23 022 | 1015 | 0.97 | 0.90-1.05 |
| Hospitals | 64 354 | 2845 | 0.99 | 0.94-1.04 |
| Hotel and other accommodation facilities | 5310 | 247 | 0.90 | 0.77-1.06 |
| IT and telecommunications | 9798 | 490 | 1.04 | 0.92-1.17 |
| Manufacture and repair of vehicles | 1236 | 61 | 1.06 | 0.75-1.49 |
| Manufacture of chemicals and pharmaceuticals | 8712 | 349 | 0.86 | 0.75-0.98 |
| Manufacture of electronic components/equipment | 4734 | 250 | 1.13 | 0.95-1.35 |
| Manufacture of Food products | 6619 | 352 | 1.11 | 0.96-1.28 |
| Manufacture of meat products | 2809 | 147 | 1.13 | 0.90-1.43 |
| Manufacture of metals and machinery | 7785 | 418 | 1.15 | 1.00-1.32 |
| Manufacture of plastic, glass and concrete | 4004 | 192 | 1.02 | 0.84-1.23 |
| Non-university education and training | 57 822 | 2374 | 0.92 | 0.87-0.97 |
| Other private offices and associations | 60 364 | 2792 | 0.95 | 0.90-0.99 |
| Public administration | 32 732 | 1420 | 0.95 | 0.89-1.02 |
| Publishing, Broadcasting and motion pictures | 9929 | 412 | 0.83 | 0.74-0.94 |
| Religious institutions and funeral activities | 2123 | 86 | 0.86 | 0.66-1.12 |
| Repair and installation of machinery and equipment | 563 | 23 | 0.86 | 0.52-1.41 |
| Research and university education | 15 419 | 604 | 0.83 | 0.75-0.91 |
| Residential centers and home care workers | 70 033 | 3645 | 1.13 | 1.08-1.18 |
| Restaurants and bars | 12 318 | 633 | 0.98 | 0.88-1.08 |
| Retail trade | 52 684 | 2759 | 1.05 | 1.00-1.11 |
| Textile and paper products | 3905 | 191 | 0.99 | 0.82-1.20 |
| Transport of gods | 7654 | 399 | 1.06 | 0.93-1.22 |
| Transport of passengers | 3418 | 159 | 0.94 | 0.77-1.14 |
| Unstated | 10 524 | 479 | 0.90 | 0.80-1.00 |
| Water supply, sewage and waste management | 697 | 40 | 1.23 | 0.78-1.94 |
| Wholesale trade | 27 991 | 1383 | 1.03 | 0.96-1.11 |
| Wood products and furniture | 2577 | 138 | 1.15 | 0.90-1.46 |

[a] Adjusted for: maternal age, parity, calendar period of birth, and quarter of conception, and sex of the child.

## S5: Sensitivity analyses

### Subdivided outcome of preterm birth intro three categories (Table S5-S6)

Table S5: Number of births (N), observed number of very preterm and moderately preterm birth cases, with occupational groups ordered by social class (1. to 4.) and alphabetically within social class. Risk rates (RR) and 99% confidence interval (CI) for very preterm and moderately preterm, compared with the average population risk.

| **Occupational group and social class** | **N total** | **Observed very preterm cases** | **Observed moderately preterm cases** | **RR[a] for very preterm** | **99% CI** | **RR[a] for moderately preterm** | **99% CI** |
|---|---|---|---|---|---|---|---|
| 1.Medical doctors, dentists, and veterinarians | 12 537 | 63 | 372 | 0.62 | 0.44-0.87 | 0.83 | 0.72-0.95 |
| 1.Professionals (physics, mathematics, engineering, architects) | 15 350 | 99 | 514 | 0.72 | 0.55-0.93 | 0.88 | 0.79-0.99 |
| 1.Professionals at academic level, not elsewhere classified | 54 556 | 392 | 1837 | 0.81 | 0.71-0.93 | 0.89 | 0.83-0.94 |
| 1.Senior officials and corporate managers | 6035 | 63 | 222 | 1.13 | 0.81-1.56 | 0.95 | 0.80-1.13 |
| 2.Associate professionals (business and administration) | 51 144 | 443 | 1882 | 0.97 | 0.86-1.10 | 0.96 | 0.90-1.02 |
| 2.Associate professionals (physics and engineering) | 10 065 | 105 | 328 | 1.16 | 0.90-1.50 | 0.86 | 0.74-0.99 |
| 2.Managers (<10 employees) | 2247 | 19 | 93 | 0.95 | 0.52-1.72 | 1.07 | 0.82-1.39 |
| 2.Psychologists and social workers | 10 062 | 84 | 360 | 0.99 | 0.75-1.33 | 0.96 | 0.84-1.11 |
| 2.Teachers in higher, secondary, vocational, and special education | 15 615 | 110 | 466 | 0.81 | 0.63-1.05 | 0.80 | 0.71-0.91 |
| 3. Associate professionals (health) | 18 653 | 159 | 693 | 1.05 | 0.85-1.29 | 1.00 | 0.91-1.11 |
| 3. Associate professionals (nursery and kindergarten) | 35 883 | 308 | 1260 | 1.01 | 0.87-1.18 | 0.94 | 0.87-1.01 |
| 3.Associate professionals (special education and care) | 12 758 | 114 | 511 | 1.00 | 0.78-1.28 | 1.02 | 0.91-1.14 |
| 3.Associate professionals, not elsewhere classified | 41 749 | 391 | 1685 | 1.09 | 0.95-1.24 | 1.02 | 0.96-1.09 |
| 3.Clerks without customer contacts | 44 230 | 408 | 1719 | 1.06 | 0.93-1.20 | 1.02 | 0.95-1.08 |
| 3.Clerks, not elsewhere classified | 13 661 | 137 | 598 | 1.13 | 0.90-1.40 | 1.11 | 1.00-1.23 |
| 3.Customer service clerks | 17 350 | 156 | 710 | 1.04 | 0.84-1.28 | 1.07 | 0.97-1.18 |
| 3.Nurses and midwives | 38 133 | 294 | 1383 | 0.96 | 0.82-1.12 | 0.99 | 0.92-1.06 |
| 3.Primary School teachers | 30 583 | 211 | 981 | 0.84 | 0.70-1.01 | 0.88 | 0.80-0.96 |
| 3.Public safety workers | 2303 | 19 | 92 | 0.91 | 0.49-1.68 | 1.01 | 0.77-1.34 |
| 3.Sales and service workers, not elsewhere classified | 1919 | 16 | 80 | 0.90 | 0.47-1.71 | 1.02 | 0.77-1.36 |
| 3.Travel attendants and related workers | 1707 | 15 | 75 | 0.91 | 0.46-1.80 | 1.09 | 0.81-1.47 |
| 4.Assembly workers | 2183 | 21 | 106 | 1.10 | 0.62-1.94 | 1.26 | 0.98-1.62 |
| 4.Childcare workers in private homes | 19 206 | 133 | 722 | 0.82 | 0.65-1.03 | 0.98 | 0.89-1.08 |
| 4.Cleaners, janitors and kitchen helpers | 17 693 | 177 | 718 | 1.08 | 0.89-1.31 | 1.05 | 0.96-1.16 |
| 4.Cooks and housekeepers | 7894 | 71 | 341 | 1.01 | 0.74-1.37 | 1.09 | 0.95-1.25 |
| 4.Drivers and manual workers in construction or manufacturing industries | 6015 | 65 | 255 | 1.18 | 0.86-1.63 | 1.06 | 0.91-1.25 |
| 4.Food and beverage production workers | 4432 | 41 | 196 | 1.03 | 0.68-1.54 | 1.11 | 0.92-1.34 |
| 4.Hairdressers, beauticians, and related workers | 7358 | 63 | 300 | 0.99 | 0.71-1.39 | 1.03 | 0.88-1.20 |
| 4.Home care workers | 26 603 | 288 | 1208 | 1.24 | 1.07-1.45 | 1.15 | 1.07-1.24 |
| 4.Nursing home workers | 20 377 | 185 | 853 | 1.06 | 0.87-1.28 | 1.09 | 0.99-1.19 |
| 4.Painters | 2277 | 17 | 81 | 0.86 | 0.45-1.62 | 0.88 | 0.66-1.19 |
| 4.Production and plant operators | 3088 | 39 | 131 | 1.39 | 0.92-2.11 | 1.08 | 0.86-1.36 |
| 4.Shop assistants | 29 683 | 288 | 1322 | 1.08 | 0.92-1.26 | 1.08 | 1.01-1.16 |
| 4.Skilled and unskilled agricultural, forest, and fishery workers | 4672 | 41 | 190 | 1.04 | 0.69-1.57 | 1.09 | 0.90-1.31 |
| 4.Skilled workers, not elsewhere classified | 3622 | 36 | 149 | 1.10 | 0.72-1.70 | 1.05 | 0.85-1.29 |
| 4.Unskilled workers NEC | 1514 | 11 | 56 | 0.73 | 0.34-1.60 | 0.89 | 0.63-1.25 |

| | | | | | | | |
|---|---|---|---|---|---|---|---|
| 4.Unstated | 49 154 | 477 | 2023 | 1.07 | 0.95-1.20 | 1.04 | 0.99-1.11 |
| 4.Waiting staff and bartenders | 2038 | 14 | 82 | 0.69 | 0.35-1.39 | 0.92 | 0.69-1.21 |

[a] Adjusted for: maternal age, BMI, smoking, parity, marital status, country of origin, calendar period of birth, and quarter of conception, and sex of the child. NEC=Not elsewhere classified. Job group classification by social class is described in Supplementary, section S6.

| Table S6: Number of births (N), observed number of very preterm and moderately preterm birth cases on maternal industrial group, ordered alphabetically. Risk rates (RR1) and 99% confidence interval (CI) for very preterm and moderately preterm, respectively, compared with the average population risk. | | | | | | | |
|---|---|---|---|---|---|---|---|
| **Industrial group** | **N total** | **Observed very preterm cases** | **Observed moderately preterm cases** | **RR[a] for very preterm** | **99% CI** | **RR[a] for moderately preterm** | **99% CI** |
| Agriculture, forestry and fishing | 3206 | 26 | 157 | 0.94 | 0.56-1.57 | 1.25 | 1.01-1.53 |
| Building completion and finishing | 3874 | 30 | 164 | 0.85 | 0.53-1.38 | 1.03 | 0.84-1.26 |
| Civil engineering | 792 | 7 | 26 | 1.01 | 0.38-2.68 | 0.86 | 0.52-1.42 |
| Cleaning industry | 14 108 | 152 | 553 | 1.10 | 0.89-1.36 | 0.98 | 0.88-1.09 |
| Construction and demolition of buildings | 1854 | 14 | 66 | 0.87 | 0.43-1.75 | 0.94 | 0.68-1.29 |
| Culture and sports | 8953 | 77 | 335 | 0.92 | 0.69-1.24 | 0.93 | 0.81-1.07 |
| Daycare (all ages) | 62 721 | 511 | 2323 | 0.94 | 0.84-1.06 | 0.97 | 0.92-1.02 |
| Defense, security and justice activities | 7821 | 73 | 272 | 1.02 | 0.75-1.38 | 0.88 | 0.75-1.03 |
| Energy, mining and quarrying | 1683 | 13 | 45 | 0.90 | 0.44-1.87 | 0.72 | 0.49-1.07 |
| Finance and insurance activities | 21 232 | 178 | 821 | 0.92 | 0.76-1.13 | 0.98 | 0.89-1.08 |
| Hairdressing and other personal service activities | 8969 | 72 | 349 | 0.84 | 0.62-1.15 | 0.91 | 0.79-1.05 |
| Health practitioners and veterinarians | 23 022 | 175 | 840 | 1.00 | 0.82-1.22 | 1.04 | 0.95-1.14 |
| Hospitals | 64 354 | 490 | 2355 | 1.00 | 0.89-1.13 | 1.04 | 0.99-1.10 |
| Hotel and other accommodation facilities | 5310 | 41 | 206 | 0.77 | 0.51-1.15 | 0.90 | 0.75-1.08 |
| IT and telecommunications | 9798 | 87 | 403 | 0.99 | 0.75-1.31 | 1.07 | 0.94-1.22 |
| Manufacture and repair of vehicles | 1236 | 13 | 48 | 1.16 | 0.57-2.39 | 0.99 | 0.68-1.44 |
| Manufacture of chemicals and pharmaceuticals | 8712 | 62 | 287 | 0.84 | 0.60-1.17 | 0.91 | 0.78-1.06 |
| Manufacture of electronic components/equipment | 4734 | 42 | 208 | 0.99 | 0.66-1.49 | 1.14 | 0.95-1.36 |
| Manufacture of Food products | 6619 | 62 | 290 | 1.02 | 0.73-1.42 | 1.09 | 0.93-1.27 |
| Manufacture of meat products | 2809 | 26 | 121 | 1.00 | 0.60-1.68 | 1.09 | 0.86-1.37 |
| Manufacture of metals and machinery | 7785 | 86 | 332 | 1.24 | 0.94-1.64 | 1.11 | 0.96-1.28 |
| Manufacture of plastic, glass and concrete | 4004 | 46 | 146 | 1.26 | 0.86-1.86 | 0.95 | 0.76-1.17 |
| Non-university education and training | 57 822 | 431 | 1943 | 0.96 | 0.84-1.09 | 0.96 | 0.90-1.02 |
| Other private offices and associations | 60 364 | 510 | 2282 | 0.95 | 0.84-1.06 | 0.98 | 0.92-1.03 |
| Public administration | 32 732 | 263 | 1157 | 1.00 | 0.85-1.17 | 0.99 | 0.91-1.07 |
| Publishing, Broadcasting and motion pictures | 9929 | 79 | 333 | 0.84 | 0.63-1.13 | 0.85 | 0.73-0.98 |
| Religious institutions and funeral activities | 2123 | 17 | 69 | 0.96 | 0.51-1.81 | 0.89 | 0.65-1.23 |

| | | | | | | | |
|---|---|---|---|---|---|---|---|
| Repair and installation of machinery and equipment | 563 | 5 | 18 | 0.97 | 0.31-3.11 | 0.82 | 0.45-1.51 |
| Research and university education | 15 419 | 106 | 498 | 0.84 | 0.65-1.09 | 0.90 | 0.80-1.01 |
| Residential centers and home care workers | 70 033 | 698 | 2947 | 1.10 | 1.00-1.21 | 1.05 | 1.00-1.10 |
| Restaurants and bars | 12 318 | 105 | 528 | 0.86 | 0.66-1.10 | 0.98 | 0.88-1.10 |
| Retail trade | 52 684 | 497 | 2262 | 1.00 | 0.89-1.12 | 1.02 | 0.96-1.07 |
| Textile and paper products | 3905 | 32 | 159 | 0.93 | 0.59-1.47 | 1.05 | 0.86-1.29 |
| Transport of gods | 7654 | 102 | 297 | 1.36 | 1.05-1.76 | 0.95 | 0.82-1.11 |
| Transport of passengers | 3418 | 31 | 128 | 0.90 | 0.56-1.43 | 0.89 | 0.71-1.12 |
| Unstated | 10 524 | 98 | 381 | 0.99 | 0.77-1.29 | 0.90 | 0.79-1.03 |
| Water supply, sewage and waste management | 697 | 9 | 31 | 1.45 | 0.61-3.44 | 1.15 | 0.73-1.83 |
| Wholesale trade | 27 991 | 282 | 1101 | 1.11 | 0.95-1.30 | 1.01 | 0.93-1.09 |
| Wood products and furniture | 2577 | 25 | 113 | 1.12 | 0.66-1.89 | 1.13 | 0.88-1.44 |

[a] Adjusted for: maternal age, BMI, smoking, parity, marital status, country of origin, calendar period of birth, quarter of conception, and socioeconomic status, and sex of the child.

## Restriction to firstborns (Table S7-S8)

Table S7. Analyses restricted to first-born children in the study period, with occupational groups, ordered by social class (1. to 4.) and alphabetically within social class. Number of births (N), observed number of cases of preterm birth, and adjusted risk ratios (RR) with 99% confidence interval (CI). N=428 639.

| | | | Compared with the average risk | |
|---|---|---|---|---|
| **Occupational group and social class** | **N** | **Observed cases** | **RR[a]** | **99% CI** |
| 1.Medical doctors, dentists, and veterinarians | 7144 | 305 | 0.81 | (0.69-0.93) |
| 1.Professionals (physics, mathematics, engineering, architects) | 9441 | 439 | 0.84 | (0.74-0.94) |
| 1.Professionals at academic level, not elsewhere classified | 34 589 | 1664 | 0.87 | (0.82-0.93) |
| 1.Senior officials and corporate managers | 3713 | 200 | 0.95 | (0.79-1.14) |
| 2.Associate professionals (business and administration) | 32 882 | 1754 | 0.96 | (0.90-1.02) |
| 2.Associate professionals (physics and engineering) | 6585 | 353 | 0.98 | (0.85-1.12) |
| 2.Managers (<10 employees) | 1307 | 77 | 1.01 | (0.74-1.35) |
| 2.Psychologists and social workers | 5834 | 314 | 0.97 | (0.83-1.12) |
| 2.Teachers in higher, secondary, vocational, and special education | 9378 | 411 | 0.80 | (0.70-0.91) |
| 3. Associate professionals (health) | 11 559 | 642 | 1.03 | (0.93-1.14) |
| 3.Associate professionals (nursery and kindergarten) | 22 086 | 1194 | 0.99 | (0.92-1.07) |
| 3.Associate professionals (special education and care) | 8383 | 504 | 1.06 | (0.95-1.19) |
| 3.Associate professionals, not elsewhere classified | 31 289 | 1693 | 1.01 | (0.95-1.07) |
| 3.Clerks without customer contacts | 28 451 | 1614 | 1.03 | (0.96-1.10) |
| 3.Clerks, not elsewhere classified | 10 450 | 617 | 1.11 | (1.00-1.23) |
| 3.Customer service clerks | 11 141 | 679 | 1.10 | (0.99-1.21) |
| 3.Nurses and midwives | 22 960 | 1220 | 0.99 | (0.92-1.07) |
| 3.Primary School teachers | 18 179 | 875 | 0.90 | (0.82-0.98) |
| 3.Public safety workers | 1469 | 88 | 1.05 | (0.78-1.37) |
| 3.Sales and service workers, not elsewhere classified | 1460 | 79 | 0.98 | (0.72-1.31) |
| 3.Travel attendants and related workers | 1240 | 75 | 1.08 | (0.79-1.45) |
| 4.Assembly workers | 1584 | 99 | 1.19 | (0.90-1.53) |
| 4.Childcare workers in private homes | 13 276 | 664 | 0.94 | (0.85-1.04) |
| 4.Cleaners, janitors and kitchen helpers | 13 860 | 742 | 1.05 | (0.95-1.15) |
| 4.Cooks and housekeepers | 5523 | 318 | 1.04 | (0.90-1.20) |
| 4.Drivers and manual workers in construction or manufacturing industries | 4422 | 267 | 1.11 | (0.94-1.30) |
| 4.Food and beverage production workers | 3210 | 186 | 1.07 | (0.88-1.29) |
| 4.Hairdressers, beauticians, and related workers | 5020 | 293 | 1.05 | (0.89-1.21) |
| 4.Home care workers | 17947 | 1155 | 1.17 | (1.09-1.27) |
| 4.Nursing home workers | 13 102 | 786 | 1.09 | (1.00-1.20) |
| 4.Painters | 1594 | 82 | 0.92 | (0.68-1.22) |
| 4.Production and plant operators | 2196 | 131 | 1.10 | (0.87-1.37) |
| 4.Shop assistants | 21 488 | 1306 | 1.07 | (1.00-1.15) |
| 4.Skilled and unskilled agricultural, forest, and fishery workers | 3168 | 181 | 1.10 | (0.90-1.33) |
| 4.Skilled workers, not elsewhere classified | 2584 | 153 | 1.09 | (0.87-1.33) |
| 4.Unskilled workers NEC | 1240 | 59 | 0.88 | (0.61-1.22) |
| 4.Unstated | 37 222 | 2052 | 1.04 | (0.98-1.10) |
| 4.Waiting staff and bartenders | 1663 | 86 | 0.90 | (0.67-1.18) |

[a] Adjusted for: maternal age, BMI, smoking, parity, marital status, country of origin, calendar period of birth, and quarter of conception, and sex of the child. NEC=Not elsewhere classified. Job group classification by social class is described in Supplementary, section S6.

Table S8. Analyses restricted to only first-born children in study period, with industrial groups ordered alphabetically. Number of births (N), observed number of cases of preterm birth, and adjusted risk ratios (RR) with 99% confidence interval (CI). N=428 639.

| | | | **Compared with the average risk** | |
|---|---|---|---|---|
| **Industrial group** | **N** | **Observed cases** | **RR[a]** | **99% CI** |
| Agriculture, forestry and fishing | 2422 | 154 | 1.22 | (0.98-1.50) |
| Building completion and finishing | 2665 | 162 | 1.06 | (0.86-1.29) |
| Civil engineering | 577 | 26 | 0.86 | (0.49-1.39) |
| Cleaning industry | 10 951 | 587 | 1.01 | (0.90-1.12) |
| Construction and demolition of buildings | 1167 | 62 | 0.98 | (0.69-1.34) |
| Culture and sports | 6473 | 333 | 0.92 | (0.79-1.06) |
| Daycare (all ages) | 40 572 | 2189 | 0.98 | (0.93-1.04) |
| Defense, security and justice activities | 5045 | 258 | 0.89 | (0.76-1.05) |
| Energy, mining and quarrying | 1029 | 42 | 0.76 | (0.49-1.11) |
| Finance and insurance activities | 12 973 | 764 | 1.02 | (0.92-1.12) |
| Hairdressing and other personal service activities | 6200 | 342 | 0.93 | (0.81-1.07) |
| Health practitioners and veterinarians | 14 108 | 757 | 1.06 | (0.97-1.17) |
| Hospitals | 39 319 | 2098 | 1.05 | (0.99-1.11) |
| Hotel and other accommodation facilities | 4045 | 203 | 0.87 | (0.72-1.04) |
| IT and telecommunications | 6454 | 359 | 1.01 | (0.88-1.16) |
| Manufacture and repair of vehicles | 826 | 50 | 1.09 | (0.73-1.55) |
| Manufacture of chemicals and pharmaceuticals | 5492 | 261 | 0.90 | (0.77-1.06) |
| Manufacture of electronic components/equipment | 3241 | 201 | 1.15 | (0.95-1.37) |
| Manufacture of Food products | 4651 | 292 | 1.12 | (0.96-1.30) |
| Manufacture of meat products | 2052 | 115 | 1.04 | (0.81-1.32) |
| Manufacture of metals and machinery | 5178 | 316 | 1.12 | (0.96-1.29) |
| Manufacture of plastic, glass and concrete | 2750 | 150 | 1.01 | (0.81-1.24) |
| Non-university education and training | 35 572 | 1768 | 0.98 | (0.92-1.04) |
| Other private offices and associations | 41 222 | 2164 | 0.96 | (0.91-1.01) |
| Public administration | 20 065 | 1037 | 0.99 | (0.91-1.07) |
| Publishing, Broadcasting and motion pictures | 7011 | 321 | 0.82 | (0.71-0.95) |
| Religious institutions and funeral activities | 1365 | 57 | 0.81 | (0.56-1.13) |
| Repair and installation of machinery and equipment | 391 | 15 | 0.70 | (0.32-1.32) |
| Research and university education | 10 060 | 466 | 0.90 | (0.79-1.01) |
| Residential centers and home care workers | 46 700 | 2830 | 1.07 | (1.02-1.12) |
| Restaurants and bars | 9904 | 541 | 0.96 | (0.85-1.07) |
| Retail trade | 38 519 | 2247 | 1.00 | (0.95-1.06) |
| Textile and paper products | 2702 | 143 | 0.99 | (0.79-1.23) |
| Transport of gods | 5367 | 324 | 1.06 | (0.91-1.22) |
| Transport of passengers | 2469 | 128 | 0.89 | (0.70-1.12) |
| Unstated | 8443 | 404 | 0.91 | (0.80-1.04) |
| Water supply, sewage and waste management | 466 | 31 | 1.21 | (0.72-1.89) |
| Wholesale trade | 18 418 | 1046 | 1.02 | (0.94-1.10) |
| Wood products and furniture | 1775 | 114 | 1.19 | (0.92-1.51) |

[a] Adjusted for: maternal age, BMI, smoking, parity, marital status, country of origin, calendar period of birth, quarter of conception, and socioeconomic status, and sex of the child.

# Further adjustment for household income (Table S9-S10)

Table S9: Main model with further adjustment for household income, with occupational groups ordered by social class (1. to 4.) and alphabetically within social class. Number of births (N), observed number of cases of preterm birth, and adjusted risk ratios (RR) with 99% confidence interval (CI). N=644 349.

| Occupational group and social class | N | Observed cases | Compared with the average risk | |
|---|---|---|---|---|
| | | | **RR[a]** | **99% CI** |
| 1.Medical doctors, dentists, and veterinarians | 12 537 | 435 | 0.81 | 0.71-0.92 |
| 1.Professionals (physics, mathematics, engineering, architects) | 15 350 | 613 | 0.87 | 0.78-0.97 |
| 1.Professionals at academic level, not elsewhere classified | 54 556 | 2229 | 0.88 | 0.84-0.93 |
| 1.Senior officials and corporate managers | 6035 | 285 | 1.00 | 0.86-1.17 |
| 2.Associate professionals (business and administration) | 51 144 | 2325 | 0.97 | 0.92-1.02 |
| 2.Associate professionals (physics and engineering) | 10 065 | 433 | 0.92 | 0.81-1.04 |
| 2.Managers (<10 employees) | 2247 | 112 | 1.05 | 0.82-1.34 |
| 2.Psychologists and social workers | 10 062 | 444 | 0.97 | 0.86-1.10 |
| 2.Teachers in higher, secondary, vocational, and special education | 15 615 | 576 | 0.81 | 0.73-0.91 |
| 3.Associate professionals (health) | 18 653 | 852 | 1.01 | 0.92-1.11 |
| 3.Associate professionals (nursery and kindergarten) | 35 883 | 1568 | 0.95 | 0.88-1.01 |
| 3.Associate professionals (special education and care) | 12 758 | 625 | 1.01 | 0.91-1.12 |
| 3.Associate professionals, not elsewhere classified | 41 749 | 2076 | 1.04 | 0.98-1.10 |
| 3.Clerks without customer contacts | 44 230 | 2127 | 1.02 | 0.97-1.08 |
| 3.Clerks, not elsewhere classified | 13 661 | 735 | 1.11 | 1.01-1.22 |
| 3.Customer service clerks | 17 350 | 866 | 1.07 | 0.97-1.17 |
| 3.Nurses and midwives | 38 133 | 1677 | 0.98 | 0.92-1.05 |
| 3.Primary School teachers | 30 583 | 1192 | 0.87 | 0.80-0.94 |
| 3.Public safety workers | 2303 | 111 | 0.99 | 0.77-1.28 |
| 3.Sales and service workers, not elsewhere classified | 1919 | 96 | 1.00 | 0.77-1.30 |
| 3.Travel attendants and related workers | 1707 | 90 | 1.06 | 0.80-1.39 |
| 4.Assembly workers | 2183 | 127 | 1.22 | 0.97-1.53 |
| 4.Childcare workers in private homes | 19 206 | 855 | 0.95 | 0.87-1.04 |
| 4.Cleaners, janitors and kitchen helpers | 17 693 | 895 | 1.05 | 0.97-1.15 |
| 4.Cooks and housekeepers | 7894 | 412 | 1.07 | 0.94-1.22 |
| 4.Drivers and manual workers in construction or manufacturing industries | 6015 | 320 | 1.08 | 0.94-1.25 |
| 4.Food and beverage production workers | 4432 | 237 | 1.09 | 0.92-1.29 |
| 4.Hairdressers, beauticians, and related workers | 7358 | 363 | 1.02 | 0.89-1.17 |
| 4.Home care workers | 26 603 | 1496 | 1.16 | 1.09-1.25 |
| 4.Nursing home workers | 20 377 | 1038 | 1.08 | 0.99-1.17 |
| 4.Painters | 2277 | 98 | 0.88 | 0.67-1.15 |
| 4.Production and plant operators | 3088 | 170 | 1.13 | 0.93-1.38 |
| 4.Shop assistants | 29 683 | 1610 | 1.08 | 1.01-1.15 |
| 4.Skilled and unskilled agricultural, forest, and fishery workers | 4672 | 231 | 1.08 | 0.91-1.28 |
| 4.Skilled workers, not elsewhere classified | 3622 | 185 | 1.05 | 0.87-1.27 |
| 4.Unskilled workers NEC | 1514 | 67 | 0.87 | 0.64-1.18 |
| 4.Unstated | 49 154 | 2500 | 1.05 | 1.00-1.11 |
| 4.Waiting staff and bartenders | 2038 | 96 | 0.88 | 0.68-1.14 |

[a] Adjusted for: maternal age, BMI, smoking, parity, marital status, country of origin, calendar period of birth, and quarter of conception, and sex of the child. NEC=Not elsewhere classified. Job group classification by social class is described in Supplementary, section S6.

Table S10: Main model with further adjustment for household income, with industrial groups ordered alphabetically. Number of births (N), observed number of cases of preterm birth, and adjusted risk ratios (RR) with 99% confidence interval (CI). N=644 349.

| | | | Compared with the average risk | |
|---|---|---|---|---|
| **Industrial group** | **N** | **Observed cases** | **RR[a]** | **99% CI** |
| Agriculture, forestry and fishing | 3206 | 183 | 1.19 | 0.98-1.44 |
| Building completion and finishing | 3874 | 194 | 0.99 | 0.82-1.20 |
| Civil engineering | 792 | 33 | 0.89 | 0.57-1.39 |
| Cleaning industry | 14 108 | 705 | 1.01 | 0.91-1.11 |
| Construction and demolition of buildings | 1854 | 80 | 0.93 | 0.69-1.24 |
| Culture and sports | 8953 | 412 | 0.93 | 0.82-1.06 |
| Daycare (all ages) | 62 721 | 2834 | 0.96 | 0.92-1.01 |
| Defense, security and justice activities | 7821 | 345 | 0.91 | 0.79-1.05 |
| Energy, mining and quarrying | 1683 | 58 | 0.77 | 0.54-1.09 |
| Finance and insurance activities | 21 232 | 999 | 0.98 | 0.90-1.07 |
| Hairdressing and other personal service activities | 8969 | 421 | 0.90 | 0.79-1.03 |
| Health practitioners and veterinarians | 23 022 | 1015 | 1.04 | 0.95-1.13 |
| Hospitals | 64 354 | 2845 | 1.04 | 0.98-1.09 |
| Hotel and other accommodation facilities | 5310 | 247 | 0.88 | 0.75-1.04 |
| IT and telecommunications | 9798 | 490 | 1.07 | 0.95-1.20 |
| Manufacture and repair of vehicles | 1236 | 61 | 1.02 | 0.73-1.42 |
| Manufacture of chemicals and pharmaceuticals | 8712 | 349 | 0.91 | 0.79-1.05 |
| Manufacture of electronic components/equipment | 4734 | 250 | 1.11 | 0.94-1.31 |
| Manufacture of Food products | 6619 | 352 | 1.07 | 0.94-1.24 |
| Manufacture of meat products | 2809 | 147 | 1.07 | 0.86-1.32 |
| Manufacture of metals and machinery | 7785 | 418 | 1.13 | 1.00-1.29 |
| Manufacture of plastic, glass and concrete | 4004 | 192 | 1.01 | 0.83-1.22 |
| Non-university education and training | 57 822 | 2374 | 0.96 | 0.91-1.01 |
| Other private offices and associations | 60 364 | 2792 | 0.98 | 0.93-1.03 |
| Public administration | 32 732 | 1420 | 0.99 | 0.92-1.06 |
| Publishing, Broadcasting and motion pictures | 9929 | 412 | 0.85 | 0.75-0.97 |
| Religious institutions and funeral activities | 2123 | 86 | 0.91 | 0.68-1.21 |
| Repair and installation of machinery and equipment | 563 | 23 | 0.85 | 0.50-1.46 |
| Research and university education | 15 419 | 604 | 0.89 | 0.80-0.99 |
| Residential centers and home care workers | 70 033 | 3645 | 1.05 | 1.01-1.10 |
| Restaurants and bars | 12 318 | 633 | 0.97 | 0.87-1.07 |
| Retail trade | 52 684 | 2759 | 1.01 | 0.97-1.07 |
| Textile and paper products | 3905 | 191 | 1.03 | 0.86-1.25 |
| Transport of gods | 7654 | 399 | 1.03 | 0.91-1.18 |
| Transport of passengers | 3418 | 159 | 0.90 | 0.73-1.11 |
| Unstated | 10 524 | 479 | 0.93 | 0.82-1.04 |
| Water supply, sewage and waste management | 697 | 40 | 1.21 | 0.81-1.81 |
| Wholesale trade | 27 991 | 1383 | 1.03 | 0.96-1.11 |
| Wood products and furniture | 2577 | 138 | 1.12 | 0.90-1.40 |

[a] Adjusted for: maternal age, BMI, smoking, parity, marital status, country of origin, calendar period of birth, quarter of conception, and socioeconomic status, and sex of the child.

## Analyses stratified by time (Table S11-S14)

Table S11: Analyses including births from April 1st 2004 to December 31st 2011, with occupational groups ordered by social class (1. to 4.) and alphabetically within social class. Number of births (N), observed number of cases of preterm birth, and adjusted risk ratios (RR) with 99% confidence interval (CI). N=347 153.

| | | | Compared with the average risk | |
|---|---|---|---|---|
| **Occupational group and social class** | **N** | **Observed cases** | **RR[a]** | **99% CI** |
| 1.Medical doctors, dentists, and veterinarians | 5603 | 204 | 0.81 | 0.68-0.98 |
| 1.Professionals (physics, mathematics, engineering, architects) | 6733 | 278 | 0.86 | 0.74-1.01 |
| 1.Professionals at academic level, not elsewhere classified | 27 701 | 1138 | 0.87 | 0.80-0.94 |
| 1.Senior officials and corporate managers | 3302 | 146 | 0.90 | 0.73-1.12 |
| 2.Associate professionals (business and administration) | 28 295 | 1346 | 0.99 | 0.92-1.06 |
| 2.Associate professionals (physics and engineering) | 5733 | 266 | 0.97 | 0.83-1.14 |
| 2.Managers (<10 employees) | 688 | 33 | 0.98 | 0.63-1.53 |
| 2.Psychologists and social workers | 2988 | 114 | 0.82 | 0.65-1.05 |
| 2.Teachers in higher, secondary, vocational, and special education | 5517 | 213 | 0.83 | 0.70-1.00 |
| 3.Associate professionals (health) | 9755 | 456 | 1.01 | 0.90-1.15 |
| 3.Associate professionals (nursery and kindergarten) | 19 727 | 888 | 0.96 | 0.88-1.05 |
| 3.Associate professionals (special education and care) | 6902 | 364 | 1.07 | 0.94-1.23 |
| 3.Associate professionals, not elsewhere classified | 23 294 | 1157 | 1.01 | 0.94-1.09 |
| 3.Clerks without customer contacts | 22 755 | 1087 | 0.99 | 0.92-1.07 |
| 3.Clerks, not elsewhere classified | 10 873 | 581 | 1.09 | 0.98-1.21 |
| 3.Customer service clerks | 10 177 | 508 | 1.04 | 0.93-1.17 |
| 3.Nurses and midwives | 19 266 | 835 | 0.96 | 0.87-1.05 |
| 3.Primary School teachers | 16 749 | 660 | 0.86 | 0.78-0.96 |
| 3.Public safety workers | 1136 | 61 | 1.08 | 0.77-1.51 |
| 3.Sales and service workers, not elsewhere classified | 1201 | 55 | 0.89 | 0.63-1.25 |
| 3.Travel attendants and related workers | 1082 | 52 | 0.96 | 0.67-1.38 |
| 4.Assembly workers | 1773 | 102 | 1.20 | 0.93-1.55 |
| 4.Childcare workers in private homes | 11 039 | 513 | 0.99 | 0.89-1.11 |
| 4.Cleaners, janitors and kitchen helpers | 10 456 | 555 | 1.07 | 0.96-1.20 |
| 4.Cooks and housekeepers | 3997 | 214 | 1.08 | 0.91-1.29 |
| 4.Drivers and manual workers in construction or manufacturing industries | 3430 | 196 | 1.13 | 0.95-1.36 |
| 4.Food and beverage production workers | 2857 | 145 | 1.02 | 0.82-1.26 |
| 4.Hairdressers, beauticians, and related workers | 3845 | 199 | 1.05 | 0.87-1.26 |
| 4.Home care workers | 13 495 | 766 | 1.15 | 1.05-1.26 |
| 4.Nursing home workers | 10 655 | 543 | 1.06 | 0.95-1.18 |
| 4.Painters | 1254 | 45 | 0.73 | 0.49-1.08 |
| 4.Production and plant operators | 2184 | 119 | 1.10 | 0.87-1.39 |
| 4.Shop assistants | 14 688 | 797 | 1.06 | 0.97-1.16 |
| 4.Skilled and unskilled agricultural, forest, and fishery workers | 2388 | 123 | 1.10 | 0.87-1.38 |
| 4.Skilled workers, not elsewhere classified | 2373 | 119 | 1.04 | 0.82-1.32 |
| 4.Unskilled workers NEC | 996 | 47 | 0.90 | 0.62-1.30 |
| 4.Unstated | 31 029 | 1651 | 1.07 | 1.01-1.14 |
| 4.Waiting staff and bartenders | 1217 | 61 | 0.92 | 0.66-1.27 |

[a] Adjusted for: maternal age, BMI, smoking, parity, marital status, country of origin, calendar period of birth, and quarter of conception, and sex of the child. NEC=Not elsewhere classified. Job group classification by social class is described in Supplementary, section S6.

Table S12: Analyses including births from January 1st 2012 to December 31st 2018, with occupational groups ordered by social class (1. to 4.) and alphabetically within social class. Number of births (N), observed number of cases of preterm birth, and adjusted risk ratios (RR) with 99% CI. N=297 196.

| | | | Compared with the average risk | |
|---|---|---|---|---|
| **Occupational group and social class** | **N** | **Observed cases** | **RR[a]** | **99% CI** |
| 1.Medical doctors, dentists, and veterinarians | 6934 | 231 | 0.78 | 0.65-0.93 |
| 1.Professionals (physics, mathematics, engineering, architects) | 8617 | 335 | 0.85 | 0.74-0.99 |
| 1.Professionals at academic level, not elsewhere classified | 26 855 | 1091 | 0.89 | 0.82-0.96 |
| 1.Senior officials and corporate managers | 2733 | 139 | 1.10 | 0.89-1.37 |
| 2.Associate professionals (business and administration) | 22 849 | 979 | 0.93 | 0.86-1.01 |
| 2.Associate professionals (physics and engineering) | 4332 | 167 | 0.84 | 0.69-1.03 |
| 2.Managers (<10 employees) | 1559 | 79 | 1.08 | 0.81-1.45 |
| 2.Psychologists and social workers | 7074 | 330 | 1.04 | 0.90-1.20 |
| 2.Teachers in higher, secondary, vocational, and special education | 10 098 | 363 | 0.80 | 0.70-0.92 |
| 3.Associate professionals (health) | 8898 | 396 | 1.01 | 0.89-1.16 |
| 3.Associate professionals (nursery and kindergarten) | 16 156 | 680 | 0.95 | 0.86-1.05 |
| 3.Associate professionals (special education and care) | 5856 | 261 | 0.95 | 0.80-1.11 |
| 3.Associate professionals, not elsewhere classified | 18 455 | 919 | 1.07 | 0.98-1.16 |
| 3.Clerks without customer contacts | 21 475 | 1040 | 1.06 | 0.98-1.15 |
| 3.Clerks, not elsewhere classified | 2788 | 154 | 1.21 | 0.99-1.48 |
| 3.Customer service clerks | 7173 | 358 | 1.10 | 0.96-1.26 |
| 3.Nurses and midwives | 18 867 | 842 | 1.02 | 0.93-1.12 |
| 3.Primary School teachers | 13 834 | 532 | 0.89 | 0.79-0.99 |
| 3.Public safety workers | 1167 | 50 | 0.92 | 0.63-1.34 |
| 3.Sales and service workers, not elsewhere classified | 718 | 41 | 1.22 | 0.82-1.82 |
| 3.Travel attendants and related workers | 625 | 38 | 1.23 | 0.81-1.88 |
| 4.Assembly workers | 410 | 25 | 1.34 | 0.80-2.23 |
| 4.Childcare workers in private homes | 8167 | 342 | 0.91 | 0.79-1.05 |
| 4.Cleaners, janitors and kitchen helpers | 7237 | 340 | 1.04 | 0.90-1.19 |
| 4.Cooks and housekeepers | 3897 | 198 | 1.07 | 0.89-1.28 |
| 4.Drivers and manual workers in construction or manufacturing industries | 2585 | 124 | 1.02 | 0.81-1.29 |
| 4.Food and beverage production workers | 1575 | 92 | 1.24 | 0.95-1.62 |
| 4.Hairdressers, beauticians, and related workers | 3513 | 164 | 1.00 | 0.82-1.23 |
| 4.Home care workers | 13 108 | 730 | 1.20 | 1.09-1.32 |
| 4.Nursing home workers | 9722 | 495 | 1.11 | 0.99-1.25 |
| 4.Painters | 1023 | 53 | 1.08 | 0.75-1.54 |
| 4.Production and plant operators | 904 | 51 | 1.24 | 0.86-1.77 |
| 4.Shop assistants | 14 995 | 813 | 1.10 | 1.01-1.21 |
| 4.Skilled and unskilled agricultural, forest, and fishery workers | 2284 | 108 | 1.06 | 0.83-1.36 |
| 4.Skilled workers, not elsewhere classified | 1249 | 66 | 1.10 | 0.80-1.50 |
| 4.Unskilled workers NEC | 518 | 20 | 0.80 | 0.45-1.42 |
| 4.Unstated | 18 125 | 849 | 1.01 | 0.93-1.11 |
| 4.Waiting staff and bartenders | 821 | 35 | 0.82 | 0.53-1.27 |

[a] Adjusted for: maternal age, BMI, smoking, parity, marital status, country of origin, calendar period of birth, and quarter of conception, and sex of the child. NEC=Not elsewhere classified. Job group classification by social class is described in Supplementary, section S6.

Table S13: Analyses including births from April 1st 2004 to December 31st 2011, with industrial groups ordered alphabetically. Number of births (N), observed number of cases of preterm birth, and adjusted risk ratios (RR) with 99% confidence interval (CI). N=347 153.

| Industrial group | N | Observed cases | Compared with the average risk | |
|---|---|---|---|---|
| | | | RR[a] | 99% CI |
| Agriculture, forestry and fishing | 1882 | 115 | 1.25 | 0.99-1.59 |
| Building completion and finishing | 2277 | 111 | 0.96 | 0.75-1.23 |
| Civil engineering | 490 | 18 | 0.77 | 0.42-1.41 |
| Cleaning industry | 7535 | 392 | 1.02 | 0.90-1.16 |
| Construction and demolition of buildings | 975 | 35 | 0.75 | 0.48-1.15 |
| Culture and sports | 4494 | 195 | 0.86 | 0.72-1.04 |
| Daycare (all ages) | 34 789 | 1628 | 0.98 | 0.92-1.05 |
| Defense, security and justice activities | 3915 | 180 | 0.93 | 0.76-1.13 |
| Energy, mining and quarrying | 886 | 26 | 0.64 | 0.38-1.06 |
| Finance and insurance activities | 11 356 | 545 | 0.96 | 0.86-1.08 |
| Hairdressing and other personal service activities | 4521 | 223 | 0.94 | 0.79-1.12 |
| Health practitioners and veterinarians | 10 877 | 493 | 1.05 | 0.93-1.18 |
| Hospitals | 32 818 | 1463 | 1.03 | 0.96-1.11 |
| Hotel and other accommodation facilities | 2846 | 123 | 0.80 | 0.63-1.01 |
| IT and telecommunications | 5034 | 272 | 1.12 | 0.95-1.30 |
| Manufacture and repair of vehicles | 766 | 39 | 1.03 | 0.68-1.56 |
| Manufacture of chemicals and pharmaceuticals | 4496 | 187 | 0.92 | 0.76-1.11 |
| Manufacture of electronic components/equipment | 3211 | 182 | 1.17 | 0.97-1.42 |
| Manufacture of Food products | 4270 | 225 | 1.05 | 0.89-1.25 |
| Manufacture of meat products | 1715 | 84 | 0.98 | 0.74-1.30 |
| Manufacture of metals and machinery | 4833 | 264 | 1.12 | 0.96-1.32 |
| Manufacture of plastic, glass and concrete | 2582 | 122 | 0.97 | 0.77-1.22 |
| Non-university education and training | 31 554 | 1342 | 0.98 | 0.91-1.05 |
| Other private offices and associations | 31 745 | 1515 | 0.98 | 0.92-1.05 |
| Public administration | 16 240 | 720 | 0.98 | 0.89-1.08 |
| Publishing, Broadcasting and motion pictures | 5807 | 249 | 0.87 | 0.74-1.02 |
| Religious institutions and funeral activities | 1187 | 48 | 0.89 | 0.61-1.29 |
| Repair and installation of machinery and equipment | 341 | 10 | 0.61 | 0.27-1.38 |
| Research and university education | 6978 | 274 | 0.87 | 0.74-1.02 |
| Residential centers and home care workers | 37 102 | 1974 | 1.06 | 1.00-1.12 |
| Restaurants and bars | 6147 | 316 | 0.93 | 0.81-1.08 |
| Retail trade | 28 947 | 1547 | 1.02 | 0.96-1.09 |
| Textile and paper products | 2180 | 111 | 1.04 | 0.82-1.33 |
| Transport of gods | 4664 | 247 | 1.01 | 0.86-1.20 |
| Transport of passengers | 2199 | 101 | 0.88 | 0.68-1.14 |
| Unstated | 7483 | 350 | 0.92 | 0.81-1.05 |
| Water supply, sewage and waste management | 417 | 28 | 1.37 | 0.85-2.21 |
| Wholesale trade | 15 777 | 782 | 1.01 | 0.92-1.10 |
| Wood products and furniture | 1817 | 101 | 1.15 | 0.89-1.49 |

[a] Adjusted for: maternal age, BMI, smoking, parity, marital status, country of origin, calendar period of birth, quarter of conception, and socioeconomic status, and sex of the child.

Table S14: Analyses including births from January 1st 2012 to December 31st 2018, with industrial groups ordered alphabetically. Number of births (N), observed number of cases of preterm birth, and adjusted risk ratios (RR) with 99% confidence interval (CI). N=297 196.

| | | | Compared with the average risk | |
|---|---|---|---|---|
| **Industrial group** | **N** | **Observed cases** | **RR[a]** | **99% CI** |
| Agriculture, forestry and fishing | 1324 | 68 | 1.10 | 0.80-1.50 |
| Building completion and finishing | 1597 | 83 | 1.04 | 0.79-1.39 |
| Civil engineering | 302 | 15 | 1.09 | 0.57-2.11 |
| Cleaning industry | 6573 | 313 | 0.99 | 0.86-1.14 |
| Construction and demolition of buildings | 879 | 45 | 1.15 | 0.79-1.69 |
| Culture and sports | 4459 | 217 | 1.01 | 0.84-1.20 |
| Daycare (all ages) | 27 932 | 1206 | 0.95 | 0.88-1.02 |
| Defense, security and justice activities | 3906 | 165 | 0.89 | 0.73-1.10 |
| Energy, mining and quarrying | 797 | 32 | 0.92 | 0.58-1.45 |
| Finance and insurance activities | 9876 | 454 | 0.99 | 0.87-1.12 |
| Hairdressing and other personal service activities | 4448 | 198 | 0.87 | 0.72-1.05 |
| Health practitioners and veterinarians | 12 145 | 522 | 1.03 | 0.92-1.16 |
| Hospitals | 31 536 | 1382 | 1.05 | 0.98-1.13 |
| Hotel and other accommodation facilities | 2464 | 124 | 0.98 | 0.78-1.23 |
| IT and telecommunications | 4764 | 218 | 1.01 | 0.85-1.20 |
| Manufacture and repair of vehicles | 470 | 22 | 1.01 | 0.58-1.75 |
| Manufacture of chemicals and pharmaceuticals | 4216 | 162 | 0.88 | 0.72-1.09 |
| Manufacture of electronic components/equipment | 1523 | 68 | 0.99 | 0.72-1.35 |
| Manufacture of Food products | 2349 | 127 | 1.11 | 0.88-1.40 |
| Manufacture of meat products | 1094 | 63 | 1.21 | 0.87-1.67 |
| Manufacture of metals and machinery | 2952 | 154 | 1.15 | 0.94-1.42 |
| Manufacture of plastic, glass and concrete | 1422 | 70 | 1.08 | 0.80-1.48 |
| Non-university education and training | 26 268 | 1032 | 0.94 | 0.87-1.02 |
| Other private offices and associations | 28 619 | 1277 | 0.97 | 0.90-1.05 |
| Public administration | 16 492 | 700 | 1.00 | 0.91-1.10 |
| Publishing, Broadcasting and motion pictures | 4122 | 163 | 0.83 | 0.68-1.02 |
| Religious institutions and funeral activities | 936 | 38 | 0.94 | 0.62-1.44 |
| Repair and installation of machinery and equipment | 222 | 13 | 1.25 | 0.62-2.55 |
| Research and university education | 8441 | 330 | 0.91 | 0.79-1.05 |
| Residential centers and home care workers | 32 931 | 1671 | 1.05 | 0.99-1.12 |
| Restaurants and bars | 6171 | 317 | 1.00 | 0.87-1.16 |
| Retail trade | 23 737 | 1212 | 1.00 | 0.93-1.08 |
| Textile and paper products | 1725 | 80 | 1.02 | 0.77-1.36 |
| Transport of gods | 2990 | 152 | 1.07 | 0.86-1.32 |
| Transport of passengers | 1219 | 58 | 0.94 | 0.67-1.33 |
| Unstated | 3041 | 129 | 0.94 | 0.75-1.17 |
| Water supply, sewage and waste management | 280 | 12 | 0.95 | 0.46-1.98 |
| Wholesale trade | 12 214 | 601 | 1.07 | 0.96-1.19 |
| Wood products and furniture | 760 | 37 | 1.06 | 0.69-1.62 |

[a] Adjusted for: maternal age, BMI, smoking, parity, marital status, country of origin, calendar period of birth, quarter of conception, and socioeconomic status, and sex of the child.

## Analyses restricted to mothers with Denmark as country of origin (Table S15-S16)

Table S15: Analyses restricted to mothers with Denmark as country of origin, with occupational groups ordered by social class (1. to 4.) and alphabetically within social class. Number of births (N), observed number of cases of preterm birth, and adjusted risk ratios (RR) with 99% confidence interval (CI) . N=566 289.

| Occupational group and social class | N | Observed cases | Compared with the average risk RR[a] | 99% CI |
|---|---|---|---|---|
| 1.Medical doctors, dentists, and veterinarians | 10 598 | 358 | 0.77 | 0.66-0.89 |
| 1.Professionals (physics, mathematics, engineering, architects) | 12 885 | 520 | 0.86 | 0.76-0.97 |
| 1.Professionals at academic level, not elsewhere classified | 50 179 | 2079 | 0.88 | 0.83-0.93 |
| 1.Senior officials and corporate managers | 5537 | 260 | 0.98 | 0.84-1.15 |
| 2.Associate professionals (business and administration) | 47 234 | 2166 | 0.96 | 0.91-1.02 |
| 2.Associate professionals (nursery and kindergarten) | 34 073 | 1500 | 0.96 | 0.90-1.03 |
| 2.Associate professionals (physics and engineering) | 9054 | 400 | 0.94 | 0.82-1.07 |
| 2.Managers (<10 employees) | 2020 | 100 | 1.03 | 0.79-1.33 |
| 2.Psychologists and social workers | 9239 | 418 | 0.99 | 0.87-1.12 |
| 2.Teachers in higher, secondary, vocational, and special education | 13 738 | 504 | 0.80 | 0.71-0.90 |
| 3.Associate professionals (health) | 17 113 | 794 | 1.02 | 0.93-1.13 |
| 3.Associate professionals (special education and care) | 12 384 | 611 | 1.02 | 0.92-1.14 |
| 3.Associate professionals, not elsewhere classified | 35 206 | 1763 | 1.03 | 0.97-1.09 |
| 3.Clerks without customer contacts | 40 324 | 1960 | 1.03 | 0.97-1.09 |
| 3.Clerks, not elsewhere classified | 12 619 | 689 | 1.13 | 1.02-1.24 |
| 3.Customer service clerks | 16 008 | 806 | 1.07 | 0.97-1.17 |
| 3.Nurses and midwives | 36 148 | 1571 | 0.97 | 0.91-1.04 |
| 3.Primary School teachers | 29 172 | 1135 | 0.87 | 0.80-0.94 |
| 3.Public safety workers | 2245 | 111 | 1.02 | 0.79-1.32 |
| 3.Sales and service workers, not elsewhere classified | 1573 | 84 | 1.04 | 0.79-1.37 |
| 3.Travel attendants and related workers | 1434 | 73 | 1.01 | 0.74-1.37 |
| 4.Assembly workers | 1808 | 110 | 1.27 | 0.99-1.63 |
| 4.Childcare workers in private homes | 16 676 | 748 | 0.95 | 0.86-1.04 |
| 4.Cleaners, janitors and kitchen helpers | 7848 | 432 | 1.10 | 0.97-1.24 |
| 4.Cooks and housekeepers | 6566 | 342 | 1.07 | 0.93-1.23 |
| 4.Drivers and manual workers in construction,or manufacturing industries | 4561 | 253 | 1.11 | 0.94-1.31 |
| 4.Food and beverage production workers | 3649 | 194 | 1.08 | 0.89-1.30 |
| 4.Hairdressers, beauticians, and related workers | 6869 | 347 | 1.04 | 0.90-1.20 |
| 4.Home care workers | 21 924 | 1247 | 1.17 | 1.09-1.26 |
| 4.Nursing home workers | 17 813 | 906 | 1.07 | 0.98-1.17 |
| 4.Painters | 2243 | 98 | 0.89 | 0.68-1.17 |
| 4.Production and plant operators | 2541 | 149 | 1.20 | 0.97-1.49 |
| 4.Shop assistants | 26 753 | 1451 | 1.07 | 1.00-1.14 |
| 4.Skilled and unskilled agricultural, forest, and fishery workers | 3264 | 170 | 1.11 | 0.91-1.36 |
| 4.Skilled workers, not elsewhere classified | 3286 | 170 | 1.06 | 0.87-1.30 |
| 4.Unskilled workers NEC | 1053 | 51 | 0.91 | 0.64-1.31 |
| 4.Unstated | 38 882 | 2036 | 1.07 | 1.01-1.13 |
| 4.Waiting staff and bartenders | 1770 | 82 | 0.85 | 0.64-1.13 |

[a] Adjusted for: maternal age, BMI, smoking, parity, marital status, calendar period of birth, and quarter of conception, and sex of the child. NEC=Not elsewhere classified. Job group classification by social class is described in Supplementary, section S6.

Table S16: Analyses restricted to mothers with Denmark as country of origin, with industrial groups ordered alphabetically. Number of births (N), observed number of cases of preterm birth, and adjusted risk ratios (RR) with 99% confidence interval (CI). N=566 289.

| | | | Compared with the average risk | |
|---|---|---|---|---|
| **Industrial group** | **N** | **Observed cases** | **RR[a]** | **99% CI** |
| Agriculture, forestry and fishing | 2178 | 139 | **1.30** | **1.01-1.68** |
| Building completion and finishing | 3723 | 189 | 1.01 | 0.83-1.22 |
| Civil engineering | 733 | 33 | 0.95 | 0.62-1.48 |
| Cleaning industry | 5774 | 298 | 1.00 | 0.86-1.16 |
| Construction and demolition of buildings | 1640 | 68 | 0.88 | 0.66-1.19 |
| Culture and sports | 8065 | 378 | 0.94 | 0.82-1.06 |
| Daycare (all ages) | 57 392 | 2608 | 0.96 | 0.92-1.02 |
| Defense, security and justice activities | 7524 | 338 | 0.92 | 0.80-1.06 |
| Energy, mining and quarrying | 1530 | 51 | 0.73 | 0.53-1.00 |
| Finance and insurance activities | 19 509 | 929 | 0.98 | 0.89-1.07 |
| Hairdressing and other personal service activities | 8122 | 392 | 0.92 | 0.81-1.05 |
| Health practitioners and veterinarians | 20655 | 922 | 1.04 | 0.95-1.14 |
| Hospitals | 58 930 | 2599 | 1.03 | 0.98-1.09 |
| Hotel and other accommodation facilities | 3883 | 193 | 0.92 | 0.77-1.10 |
| IT and telecommunications | 8300 | 428 | 1.08 | 0.95-1.23 |
| Manufacture and repair of vehicles | 1085 | 57 | 1.08 | 0.75-1.54 |
| Manufacture of chemicals and pharmaceuticals | 7559 | 316 | 0.93 | 0.80-1.07 |
| Manufacture of electronic components/equipment | 3999 | 223 | 1.16 | 0.96-1.41 |
| Manufacture of Food products | 5857 | 319 | 1.10 | 0.94-1.28 |
| Manufacture of meat products | 1851 | 106 | 1.15 | 0.88-1.51 |
| Manufacture of metals and machinery | 6731 | 378 | 1.18 | 1.02-1.37 |
| Manufacture of plastic, glass and concrete | 3407 | 162 | 0.99 | 0.81-1.22 |
| Non-university education and training | 53 904 | 2193 | 0.95 | 0.90-1.01 |
| Other private offices and associations | 53 677 | 2524 | 0.98 | 0.93-1.03 |
| Public administration | 29 890 | 1309 | 0.99 | 0.92-1.07 |
| Publishing, Broadcasting and motion pictures | 9243 | 381 | 0.84 | 0.74-0.95 |
| Religious institutions and funeral activities | 1995 | 80 | 0.90 | 0.68-1.20 |
| Repair and installation of machinery and equipment | 484 | 21 | 0.90 | 0.53-1.53 |
| Research and university education | 12 834 | 505 | 0.88 | 0.79-0.98 |
| Residential centers and home care workers | 61 730 | 3223 | 1.05 | 1.01-1.11 |
| Restaurants and bars | 8969 | 479 | 0.96 | 0.86-1.08 |
| Retail trade | 47 360 | 2491 | 1.01 | 0.95-1.06 |
| Textile and paper products | 2876 | 143 | 1.02 | 0.82-1.27 |
| Transport of gods | 6197 | 322 | 1.01 | 0.87-1.17 |
| Transport of passengers | 3025 | 139 | 0.88 | 0.71-1.08 |
| Unstated | 7954 | 358 | 0.90 | 0.79-1.02 |
| Water supply, sewage and waste management | 642 | 39 | 1.29 | 0.81-2.05 |
| Wholesale trade | 24 756 | 1225 | 1.02 | 0.95-1.10 |
| Wood products and furniture | 2306 | 130 | 1.19 | 0.92-1.52 |

[a] Adjusted for: maternal age, BMI, smoking, parity, marital status, calendar period of birth, quarter of conception, and socioeconomic status, and sex of the child.

# S6: Job group classification by social class

Classification of job groups by social class (1-5) inspired by the Danish Economic Council of the Labour Movement's division, ordered alphabetically within classes (1).

1. Upper Class
    - Medical doctors, dentists, and veterinarians
    - Professionals (physics, mathematics, engineering and architects)
    - Professionals at academic level, not elsewhere classified (NEC)
    - Senior officials and corporate managers

2. Upper Middle Class
    - Associate professionals (business and administration)
    - Associate professionals (physics and engineering)
    - Managers (<10 employees)
    - Psychologists and social workers
    - Teachers in higher, secondary, vocational and special education

3. Middle Class
    - Associate professionals (health)
    - Associate professionals (nursery and kindergarten)
    - Associate professionals (special education and care)
    - Associate professionals, not otherwise classified
    - Clerks, not otherwise classified
    - Clerks without customer contacts
    - Customer service clerks
    - Nurses and midwives
    - Primary school teachers
    - Public safety workers
    - Sales and service workers, not otherwise classified
    - Travel attendants and related workers

4. Working Class
    - Assembly workers
    - Child care workers in private homes
    - Cleaners, janitors and kitchen helpers
    - Cooks and house keepers
    - Drivers and manual workers in construction, or manufacturing industries
    - Food and beverage industry workers
    - Hairdressers, beauticians and related workers
    - Home care workers
    - Nursing home workers
    - Painters
    - Production and plant operators
    - Shop assistants
    - Skilled and unskilled agricultural, forest and fishery workers
    - Skilled workers, not otherwise classified
    - Unskilled workers, not otherwise classified
    - Waiting staff and bartenders

5. Long-term out of the labor market

-

(1) [Juul J.S. Det danske klassesamfund. Inddeling af befolkningen i sociale klasser. Arbejderbevægelsens Erhvervsråd, København, Danmark. https://www.ae.dk/files/dokumenter/analyse/ae_inddeling-af-befolkningen-i-sociale-klasser.pdf]
